# WWP2-induced inhibition of hepatocellular carcinoma cellular senescence via the ubiquitination and degradation of p21

**DOI:** 10.1038/s41419-025-08318-0

**Published:** 2025-12-12

**Authors:** Xiaojing Chen, Jihong Wang, Zihan Yan, Xinrui Du, Zhiyao Zhang, Xin Liu, Lanxin Hu, Kun He, Jing Zhang, Yunwei Han

**Affiliations:** 1 Department of Oncology, The Affiliated Hospital of Southwest Medical University, Luzhou, China; 2 Department of Oncology, The Affiliated Traditional Chinese Medicine Hospital of Southwest Medical University, Sichuan, China; 3 Clinical Research Institute, The Affiliated Hospital of Southwest Medical University, Luzhou, China; 4 Department of Oncology, the Second Peoples’ Hospital of Yibin, Yibin, China; 5 Luzhou Traditional Chinese Medicine Oral Preparation Pilot Platform, Sichuan, China; 6 Luzhou Key Laboratory of Interdisciplinary Science in Cancer Prevention and Treatment Integrating Chinese and Western Medicine, Sichuan, China

**Keywords:** Oncogenes, Oncogenesis

## Abstract

Hepatocellular carcinoma (HCC) is a leading cause of cancer-related mortality worldwide. The E3 ubiquitin ligase WWP2 has emerged as a critical regulator of tumor pathogenesis through its modulation of substrate ubiquitination. However, its specific mechanistic role in HCC remains poorly understood. In this study, we found that WWP2 was significantly up-regulated in HCC patients and associated with poor prognosis. Lentivirus-mediated knockdown of WWP2 induced cellular senescence and suppressed proliferation in HCC cell lines. Mechanistically, co-immunoprecipitation and ubiquitination assays identified WWP2 as a novel E3 ubiquitin ligase for p21 that promotes its K48-linked ubiquitination and subsequent proteasomal degradation, consequently accelerating cellular senescence and restraining HCC progression. Notably, we further discovered that CMTM6 directly interacts with WWP2, thereby stabilizing p21 by preventing its WWP2-mediated ubiquitination. Accordingly, the senescence and proliferation arrest induced by WWP2 deficiency were partially reversed by CMTM6 knockdown but enhanced by concurrent CMTM6 overexpression. This functional interplay was corroborated in vivo, as WWP2 depletion enhanced tumor cell senescence and suppressed tumor growth, an effect that was partially rescued by concurrent CMTM6 knockdown. Taken together, our findings establish the WWP2–CMTM6–p21 axis as a pivotal regulatory mechanism of cellular senescence in HCC and shed new light on senescence-related therapeutic strategies for HCC.

## Introduction

Hepatocellular carcinoma (HCC) occupies a prominent position in the global disease burden, ranking as the sixth most common malignancy [1] and the third leading cause of cancer-related deaths [2]. Characterized by insidious onset, rapid progression, and high recurrence rates, HCC management faces limitations with conventional therapies due to therapeutic resistance and substantial drug toxicity [3, 4]. Although breakthroughs in targeted therapy and immunotherapy have been achieved, drug resistance remains a critical constraint on clinical efficacy, which underscores the urgent need for innovative therapeutic strategies.

Cellular senescence is a state of stable cell-cycle arrest triggered by various intrinsic and extrinsic stressors [5], recently established as a novel cancer hallmark [6]. Its core mechanisms involve diminished DNA repair capacity, metabolic reprogramming, epigenetic remodeling, and activation of the senescence-associated secretory phenotype (SASP) [7]. Biomarker analyses indicate that senescent cells display reduced proliferative potential, increased senescence-associated β-galactosidase (SA-β-gal) activity, and upregulation of cell-cycle inhibitors, such as p16, p21, and p53 [8, 9]. Notably, cellular senescence exerts a context-dependent dual role in tumor progression: it can activate tumor-suppressive mechanisms to exert anticancer effects, but also remodels the tumor microenvironment through SASP to enhance tumor stemness, invasion, metastasis, and immune evasion capabilities [10]. Recent studies demonstrate that selective clearance of detrimental senescent cells or combination therapy with senolytic agents can significantly enhance anti-HCC efficacy, providing novel directions for therapeutic development [11, 12].

It is well-established that the primary and most classical role of p21 is as a tumor suppressor [13, 14]. Consistent with this function, stabilizing p21 protein has been shown to exert significant anti-tumor activity in HCC [15, 16]. Along with p16^Ink4a^, p21 has served as one of the key markers guiding research on cellular senescence in vivo over the past two decades [17]. A hallmark function of p21 is its ability to induce cell cycle arrest. Specifically, p21 inhibits cyclins A, D, and E, thereby preventing cell cycle progression and ultimately leading to cellular senescence [14]. The ubiquitin–proteasome pathway is a major regulatory mechanism controlling p21 stability through targeted degradation [18]. Nevertheless, the precise regulatory mechanisms and overall impact of ubiquitin-dependent p21 regulation on cellular senescence and HCC progression remain to be elucidated.

The ubiquitin–proteasome system (UPS) is a crucial regulatory mechanism for post-translational protein modification, responsible for mediating the specific degradation of 80–90% of short-lived proteins in cells [19, 20]. A critical regulatory feature of the UPS involves the specific formation of different types of ubiquitin chains. Among characterized ubiquitin chains, K48- and K63-linked polyubiquitination represent the two canonical types that exhibit divergent functions. K48-linked chains primarily target substrates for proteasomal degradation, whereas K63-linked chains are predominantly involved in non-proteolytic processes [21, 22]. The UPS has also attracted particular attention for its role in regulating cellular senescence [23]. For instance, E3 ubiquitin ligases WDR20 and TRIM22 induce senescence and suppress proliferation in HCC cells by promoting K48-linked ubiquitination of pro-survival proteins [24, 25]. Conversely, factors like NIPSNAP1 exert anti-senescent effects through P27 pathway inhibition and ROS reduction, revealing the complex regulatory network of ubiquitination modifications in HCC senescence [26].

The E3 ubiquitin ligase WWP2 (WW domain-containing E3 ubiquitin protein ligase 2), a member of the NEDD4 family, has garnered significant attention due to its context-dependent functional heterogeneity. This protein exhibits a characteristic tertiary structure comprising an N-terminal C2 domain, a central WW domain cluster, and a C-terminal HECT catalytic domain, regulating substrate ubiquitination to influence protein stability and functional activity [27, 28]. Substantial evidence indicates that WWP2 predominantly functions as an oncogenic driver, yet may exert tumor-suppressive effects under specific conditions. Intriguingly, WWP2 exhibits a dual role as both an oncogene and tumor suppressor in HCC through substrate-specific modulation mechanisms [29]. However, the multifaceted mechanisms of WWP2 in HCC are still incompletely understood, and its potential role in regulating cellular senescence has not been explored.

This study investigated the regulatory role of WWP2 in HCC through in vitro and in vivo experiments, with a particular emphasis on senescence modulation. Mechanistic investigations revealed that p21 serves as a ubiquitination substrate of WWP2. Specifically, WWP2 orchestrates K48-linked polyubiquitination and degradation of p21, thereby inhibiting senescence and promoting HCC progression. Furthermore, we identified that CMTM6 interacts with WWP2 to antagonize WWP2-mediated ubiquitination of p21. Collectively, our findings establish the WWP2–CMTM6–p21 axis as a promising therapeutic target for senescence-targeting therapy in HCC.

## Materials and methods

### Bioinformatics analysis

RNA sequencing data from The Cancer Genome Atlas (TCGA) were downloaded from the UCSC Xena platform (https://xena.ucsc.edu/) in Transcripts Per Million (TPM) format. These data were reprocessed using the Toil pipeline to ensure consistency across different cancer types for subsequent integrative analysis. Pan-cancer expression profiles of the WWP2 gene were extracted from this dataset, followed by rigorous sample filtering: (1) Normal and tumor tissues were segregated, and (2) cancer types with fewer than three samples were excluded. The final cohort comprised 33 TCGA cancer types. Normalized expression values from tumor and adjacent normal tissues were log2-transformed (log2[TPM + 1]) prior to statistical analysis using R software (v4.2.1). Data visualization was implemented through the ggplot2 package (v3.3.6).

For HCC patients, we integrated WWP2 mRNA expression data with clinical endpoints (Overall Survival [OS] and Progression-Free Interval [PFI]) using the following analytical pipeline: The surv_cutpoint function (survminer v0.4.9) identified the WWP2 expression threshold that maximized the survival difference between high- and low-expression groups. Then, Kaplan–Meier curves were generated using survminer v0.4.9 and ggplot2 v3.4.4 to compare survival outcomes between WWP2 expression subgroups based on the established cutoff.

### Cell culture and transfection

Human HCC cell lines HuH-7 and HCCLM3 were used for functional experiments. HEK293T cells were used exclusively for lentiviral packaging and were cultured under identical conditions. Cells were cultured in Dulbecco’s Modified Eagle Medium (DMEM; Gibco, C11995500BT) supplemented with 10% fetal bovine serum (FBS; Newzerum, FBS-S500) and maintained at 37 °C under a humidified atmosphere containing 5% CO_2_. Plasmid transfections were performed using polyethylenimine transfection reagent (Vazyme, T101-01) following the manufacturer’s standardized protocol.

### Plasmids, antibodies, reagents, and chemicals

For transient expression, cDNAs encoding WWP2, CMTM6, and p21 were subcloned into the pcDNA3.1(+) vector. To generate stable overexpression cell lines, these cDNAs were subsequently cloned into the pLV3-CMV-MCS lentiviral vector. For stable knockdown, sequence-validated shRNAs targeting WWP2 or CMTM6 were ligated into the pLV3-U6-MCS-shRNA vector. Lentiviral particles were packaged using the psPAX2 and pMD2.G plasmids.

We used the following antibodies in this study: anti-WWP2 (Proteintech, 121197-1-AP), anti-p21 (Proteintech, 10355-1-AP), anti-CMTM6 (CST, 90329S; for Western blot), anti-p53 (Abmart, TA0865), anti-p16 (Proteintech, 10883-1-AP), anti-GAPDH (Proteintech, 60004-1-Ig), anti-CMTM6 (Abmart, ME6530106; for immunohistochemistry), anti-c-MYC (Huabio, 0912-2), anti-PCNA (Proteintech, 10205-2-AP), ubiquitin polyclonal antibody (Proteintech, 10201-2-AP), K48-linkage specific ubiquitin antibody (Abmart, T55964), K63-linkage specific ubiquitin antibody (Selleck, F0528), rabbit IgG control polyclonal antibody (Proteintech, 30000-0-AP), HRP-conjugated goat anti-rabbit IgG(H + L) (Proteintech, SA00001-2), HRP-conjugated affinipure goat anti-Mouse IgG(H + L) (Proteintech, SA00001-1).

For immunoprecipitation and co-immunoprecipitation assays, primary antibodies were diluted at 1:100. For immunofluorescence and immunohistochemical staining, all antibodies were used at a 1:100 dilution. For Western blotting, anti-GAPDH and secondary antibodies were diluted at 1:5000, while all other primary antibodies were diluted at 1:1000.

We used the following chemicals: MG132 (Selleck, S2619), cycloheximide (CHX; Selleck, S7418), and puromycin (Beyotime, ST551). Protein A/G magnetic beads (Beyotime, P2108) were used for immunoprecipitation. The Cell Counting Kit-8 (APExBIO, 40203ES60), Senescence β-Galactosidase Staining Kit (Beyotime, C0602), EdU Cell Proliferation Kit (Beyotime, C0071S), and Immunohistochemical Detection Kit (Proteintech, PK10006) were used according to the manufacturers’ instructions.

### RNA sequencing and data analysis

The construction of RNA libraries and transcriptome sequencing were performed by Shanghai Majorbio Biomedical Technology. The sequenced samples comprised total RNA extracted from both WWP2-knockdown HuH-7 cells and their corresponding non-targeting control cells. Polyadenylated mRNA was enriched from total RNA using Oligo(dT) magnetic beads based on A–T complementary base pairing. Fragmentation buffer was added to randomly shear mRNA into fragments of ~300 bp, optimized for next-generation sequencing platforms. First-strand cDNA was synthesized using random hexamer primers and reverse transcriptase, followed by second-strand cDNA synthesis to generate double-stranded DNA. Blunt-end repair and 3’-adenylation of the double-stranded cDNA were performed using an End Repair Mix to facilitate adapter ligation. The ligated products were then purified, size-selected, and PCR-amplified to generate the final sequencing libraries, which were sequenced on an Illumina NovaSeq 6000 platform, yielding raw FASTQ files. Differential gene expression analysis was performed using the DESeq2 package in R software, followed by KEGG pathway enrichment analysis utilizing the DAVID database.

### Lentivirus preparation infection

To establish stable knockdown cell lines, HuH-7 cells were transduced with lentiviruses expressing two distinct shRNAs targeting WWP2 (sh-WWP2-1, sh-WWP2-2) or CMTM6 (sh-CMTM6-1, sh-CMTM6-2); the target sequences are provided in Table S1. For overexpression, the full-length coding sequences of WWP2, CMTM6, and p21 were cloned into the pLV3-CMV-MCS vector. All lentiviruses were produced by co-transfecting HEK 293T cells with the respective transfer vector along with the psPAX2 packaging and pMD2.G envelope plasmids. Transduced cells were selected with 2 μg/mL puromycin for 2 weeks.

### Western blotting

Total protein extraction was performed using RIPA lysis buffer (Beyotime, P0013B) supplemented with 1% (v/v) phosphatase inhibitor cocktail (Solarbio, P1260) and 1% (v/v) protease inhibitor (Solarbio, P0100). Protein concentration was quantified using the Bradford assay kit (Beyotime, P0006) according to the manufacturer’s protocol. Subsequently, 5 × SDS loading buffer (Solarbio, R1050) was added to the protein samples, which were then denatured at 98 °C for 10 min. Electrophoretic separation was conducted on 10% SDS–polyacrylamide gels under constant voltage (60 V for the stacking gel, 120 V for the separating gel), followed by wet transfer onto PVDF membranes (Millipore, IPVH00010) using a Trans-Blot system. After blocking with protein-free rapid blocking buffer (Epizyme Biotech, WJ107) for 30 min at room temperature, membranes were incubated with primary antibodies overnight at 4 °C. Membranes were then probed with horseradish peroxidase (HRP)-conjugated secondary antibodies for 1 h at room temperature. Protein bands were visualized using an ECL chemiluminescence detection system (Proteintech, PK10001) and quantified by densitometric analysis with ImageJ software.

### Real-time quantitative polymerase chain reaction (RT-qPCR)

Total RNA was extracted from cells using AG RNAex Pro Reagent (Accurate Biotechnology, AG21101) following the manufacturer’s protocol. Subsequently, cDNA synthesis was performed using HisyGo RT Red SuperMix (Vazyme, RT101-01). RT-qPCR was conducted on the Applied Biosystems QuantStudio 5 real-time PCR System with SYBR qPCR Master Mix (Vazyme, Q312-02). GAPDH served as the endogenous control for normalization. All reactions were performed in technical triplicate. Relative gene expression levels were determined using the comparative ΔΔ^Ct^ method. The specific primer sequences used for RT-qPCR analysis were as follows: WWP2 (forward: 5’-TCAAGAACTCAGGCCACAGTG-3’, reverse: 5’-CACCAACAACGGAAGGTTCTTC-3’), p21(CDKN1A) (forward: 5’-TGTCCGTCAGAACCCATGC-3’, reverse: 5’-AAAGTCGAAGTTCCATCGCTC-3’), and GAPDH (forward: 5’-GGAGCGAGATCCCTCCAAAAT-3’, reverse: 5’-GGCTGTTGTCATACTTCTCATGG-3’).

### Human tumor specimens

Human HCC tumor specimens and corresponding nontumor specimens utilized in this study, comprising both fresh tissue samples and paraffin-embedded sections, were obtained from the Affiliated Hospital of Southwest Medical University (Luzhou, China). All specimens were collected following written informed consent for diagnostic confirmation of HCC in accordance with the World Health Organization diagnostic criteria.

### Proliferation assay

To evaluate cell proliferation, we employed three complementary assays: colony formation assay, Cell Counting Kit-8 (CCK8) assay, and 5-ethynyl-2′-deoxyuridine (EdU) staining.

For the colony formation assay, exponentially growing cells were dissociated into single-cell suspensions using 0.25% trypsin–EDTA and seeded in six-well plates at a density of 700 viable cells/well. Plates were maintained in a humidified incubator at 37 °C with 5% CO₂ for 14 days, with medium replacement every 3 days. Colonies were gently rinsed with pre-warmed PBS to remove non-adherent debris. Colonies were treated with 4% paraformaldehyde (1 mL/well) for 15 min at room temperature to preserve cellular morphology. Immersed in 0.5% crystal violet for 10 min to visualize nuclei, followed by three deionized water washes to eliminate unbound dye. Air-dried plates were photographed under standardized lighting. Colonies containing ≥50 cells were manually enumerated.

For the CCK8 assay, cells were resuspended in fresh cell culture medium and counted using a cell counting plate. A total of 3 × 10³ cells per well were seeded in a 96-well plate, with culture medium alone serving as the blank control. Cell viability was assessed at the given time points. Two hours before analysis, 10 μL of CCK8 reagent was added to each well. Optical density (OD) values were measured at 450 nm using a multimode microplate reader (Agilent, Synergy H1).

For EdU staining, cells were seeded in a 24-well plate at a density of 3 × 10⁵ cells per well and cultured for 48 h. Subsequently, the cells were cultured in fresh DMEM medium containing EdU (10 μmol/L) and 10% FBS for an additional 2 h. Standard staining procedures were then followed according to the manufacturer’s instructions. After staining with 4′,6-diamidino-2-phenylindole (DAPI), coverslips were mounted with an anti-fluorescence quencher. Images were captured using an Olympus IX73 inverted fluorescence microscope. The percentage of EdU-positive cells was calculated: EdU-positive rate = (EdU-positive cells/Total cells) × 100%.

### Cell cycle analysis

Cell cycle distribution was analyzed using the Cell Cycle and Apoptosis Analysis Kit (Beyotime, C1052). HuH-7 cells were seeded in six-well plates at a density of 2 × 10^5^ cells/well. At 60% confluence, cells were harvested by trypsinization, washed twice with ice-cold PBS, and fixed in 70% ethanol (pre-chilled to −20 °C) overnight at 4 °C. Fixed cells were pelleted by centrifugation (300 × *g*, 5 min), rehydrated in PBS, then treated with RNase A (to degrade RNA and avoid interference) and propidium iodide (PI, for DNA staining) at 37 °C for 30 min in the dark. The cell cycle was detected using flow cytometry. Data analysis was performed using FlowJo software (BD Biosciences) to quantify G0/G1, S, and G2/M phase distributions.

### Senescence-associated β-Galactosidase staining

Cellular senescence was assessed by SA-β-gal staining per the manufacturer’s protocol (Senescence β-Galactosidase Staining Kit). SA-β-gal-positive cells, identified by perinuclear blue precipitates, were counted to determine the senescence index: (SA-β-gal-positive cells/Total cells)×100%. In tumor tissues, positive areas were quantified via threshold-based pixel density analysis (ImageJ). For each group, three independent replicates were analyzed, with five random, non-overlapping fields evaluated per replicate.

### Immunofluorescence (IF) analysis

Following distinct experimental treatments, HuH-7 cells were fixed with 4% paraformaldehyde fix solution (Biosharp, BL539A) for 15 min at room temperature. Permeabilization was performed using 0.2% Triton X-100 in PBS for 10 min, followed by blocking with 5% bovine serum albumin (BSA; Shanghai Yuanye Bio-Technology, R21514) for 30 min. Primary antibodies were applied and incubated at 4 °C overnight in a humidified chamber. After three washes with PBS containing 0.1% Tween-20 (PBST), the coverslips were incubated with species-matched secondary antibodies for 1 h at room temperature, protected from light. Triple immunofluorescence staining was conducted according to the manufacturer’s protocol for the TSA fluorescence triple staining kit (AiFang Biological, AFIHC024). Finally, nuclei were counterstained with DAPI for 5 min, and coverslips were mounted using antifade medium. Additionally, phalloidin (Beyotime, C2209S) was used to visualize F-actin and observe cell morphology. Fluorescence images were acquired using an inverted fluorescence microscope (Olympus Corporation, IX73).

### Co-immunoprecipitation assay

Cells were cultured in 10 cm dishes until reaching 80% confluency before harvesting. For protein extraction, ice-cold NP-40 lysis buffer (Solarbio, N8032) supplemented with protease and phosphatase inhibitors was added to the cell pellets, followed by a 30 min incubation on ice with periodic vortexing. The lysates were then clarified by centrifugation at 12,000 rpm for 10 min at 4 °C. To prepare the input samples, 80 μL of the supernatant was mixed with 20 μL of 5×SDS loading buffer and denatured at 98 °C for 10 min. The remaining lysate was used for immunoprecipitation by incubating with either target-specific primary antibodies or isotype-matched control IgG overnight at 4 °C with constant rotation. Antigen-antibody complexes were captured using Protein A/G magnetic beads through a 1-h incubation at room temperature with gentle agitation. The beads were then washed three times (5 min each) with chilled NP-40 buffer to remove nonspecific binding. Bound proteins were eluted in 1× SDS loading buffer by thermal denaturation at 98 °C for 10 min. The denatured samples were subsequently resolved by SDS–PAGE and analyzed via immunoblotting.

### Cycloheximide (CHX) chase experiment

To assess the degradation kinetics of endogenous p21 protein, cells were seeded at equal densities in six-well plates and treated with CHX (100 μg/mL) to inhibit de novo protein synthesis. CHX was administered for increasing time intervals to establish a time-dependent degradation profile. Following treatment, proteins were harvested using RIPA lysis buffer (Beyotime, P0013B) supplemented with protease/phosphatase inhibitors. Lysates were mixed with 5× SDS loading buffer and denatured by heating at 98 °C for 10 min to ensure complete unfolding of protein complexes. The denatured samples were resolved by SDS–PAGE and analyzed via immunoblotting to quantify the kinetics of p21 degradation.

### Ubiquitination assay

To analyze p21 ubiquitination, cells were pretreated with the proteasome inhibitor MG132 (10 μM). Proteins were extracted using NP-40 lysis buffer (Solarbio, N8032) supplemented with a cocktail of protease and phosphatase inhibitors. For input control preparation, 80 μL of the supernatant was mixed with 20 μL of 5× SDS loading buffer and denatured at 98 °C for 10 min. The remaining lysates were immunoprecipitated with an anti-p21 antibody overnight at 4 °C, then subsequently incubated with Protein A/G magnetic beads. Immunoprecipitated complexes were denatured in 1× SDS loading buffer at 98 °C for 10 min, resolved by SDS–PAGE, and followed by ubiquitination analysis via immunoblotting.

### Tumor xenograft experiments

Male BALB/c nude mice (4 weeks old, weighing 16-20 g) were procured from Chongqing Tengxin Biotechnology and acclimatized for 7 days under specific pathogen-free (SPF) conditions with a 12/12 h light/dark cycle, temperature of 20–22 °C, humidity of 50–60%, and ad libitum access to autoclaved rodent chow and UV-sterilized water. Following computer-generated randomization, 18 weight-matched mice were stratified into three experimental cohorts (*n* = 6/group). For tumor xenograft establishment, single-cell suspensions (1 × 10⁷ cells/mL in PBS) with >95% viability (as determined by trypan blue exclusion) were prepared through enzymatic dissociation and implanted subcutaneously into the right axilla using an insulin syringe. Tumor growth was monitored every three days using digital vernier calipers, with tumor volume (TV) calculated based on the modified ellipsoid formula: TV = 0.5 × length × width². In accordance with the IACUC-approved animal protocol, mice were euthanized by cervical dislocation. Excised tumors were immediately weighed, with parallel samples either fixed in 4% paraformaldehyde (24 h) for histopathology or snap-frozen in liquid nitrogen for Frozen section or molecular analyses. For the animal studies, investigators were blinded to the group allocation during the tumor measurement and data analysis phases.

### H&E and immunohistochemical staining

H&E staining was performed to observe the pathological features of tumor tissues. Briefly, 4 μm-thick paraffin-embedded sections were deparaffinized in xylene and rehydrated through a graded ethanol series. The sections were then stained with hematoxylin for nuclei, followed by counterstaining with eosin for the cytoplasm. Finally, the sections were dehydrated, cleared in xylene, and mounted with a coverslip for microscopic examination.

The paraffin-embedded tissue sections were processed following the protocol of the immunohistochemical detection kit. Initially, the sections were deparaffinized in xylene. Antigen retrieval was then performed using citric acid-based retrieval solution under heated conditions. Endogenous peroxidase activity was quenched to prevent nonspecific staining. Sections were subsequently blocked with 5% BSA to reduce background staining. Primary antibody incubation was carried out overnight at optimal conditions, followed by a 1 h incubation with the appropriate secondary antibody. Chromogenic detection was achieved using DAB substrate, and nuclei were counterstained with hematoxylin. Finally, the sections were dehydrated, mounted, and examined under a light microscope for analysis.

### Statistics analysis

Each experiment included at least three independent biological replicates. The normality of data distribution was assessed using the Shapiro–Wilk test. Homogeneity of variances was verified using the Brown–Forsythe test (for ANOVA) or *F*-test (for *t*-test). For comparisons between two unpaired groups, a two-tailed unpaired Student’s *t*-test was used if data passed both normality and homogeneity tests; otherwise, the Mann–Whitney *U* test was applied. For comparisons between two paired groups, a paired Student’s *t*-test was used for normally distributed data; otherwise, the Wilcoxon signed-rank test was applied. For comparisons among multiple groups, one-way ANOVA with Bonferroni’s post-hoc test was used if data met parametric assumptions; otherwise, the Kruskal–Wallis test with Dunn’s post-hoc test was applied. Correlations were analyzed using Spearman’s rank correlation coefficient. The data are presented as mean ± standard error of the mean (SEM). Differences were considered statistically significant at *P* < 0.05. No statistical method was used to predetermine sample size for animal studies; sample sizes were chosen based on established experimental models in the field. All statistical analyses were performed using GraphPad Prism software (version 9.0.0).

## Results

### WWP2 is highly expressed and associated with poor prognosis in HCC patients

**Fig. 1:**
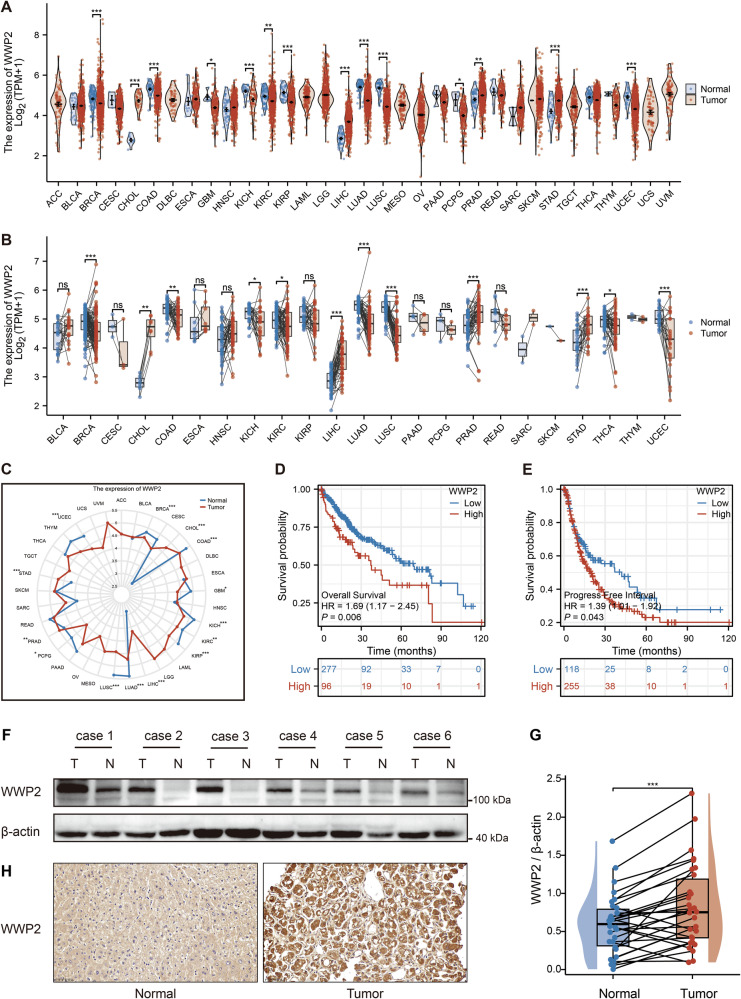
WWP2 expression patterns and clinical significance in HCC. **A** The violin plot displays the mRNA expression levels of WWP2 in tumor tissues and adjacent normal tissues across 33 cancer types from the TCGA database. **B** The scatter plot combined with a box plot illustrates the distribution of WWP2 mRNA expression in tumor tissues and their paired adjacent normal tissues across 23 cancer types. **C** Radar plot displaying the distribution patterns and statistical differences of WWP2 expression across multiple tumor tissues and their adjacent normal tissues. **D** Kaplan–Meier survival analysis of WWP2 expression levels in relation to patient overall survival (OS). **E** Kaplan–Meier survival analysis of WWP2 expression levels in relation to patient progression-free interval (PFI). **F** Representative Western blot analysis of WWP2 protein expression in 30 paired HCC tumor (T) and adjacent non-tumor (N) tissues. β-actin serves as a loading control. **G** Quantitative analysis of WWP2 protein levels (normalized to β-actin) demonstrating significant upregulation in HCC tissues versus paired normal controls. **H** Representative IHC staining images illustrating differential WWP2 expression patterns between HCC tumor tissues and their adjacent normal tissues (scale bars: 50 μm). Data presented as mean ± SEM; *P < 0.05, **P < 0.01, ***P < 0.001; ns, not significant.

Previous studies have shown that WWP2 exhibits either oncogenic or tumor-suppressive effects depending on its regulated substrates in tumors [28]. To comprehensively investigate the expression profile of WWP2, first, we analyzed its expression levels across 33 tumor types using the TCGA database. The results revealed that compared with normal tissues, WWP2 was significantly up-regulated in four tumor types: liver hepatocellular carcinoma (LIHC), cholangiocarcinoma (CHOL), prostate cancer (PRAD), and stomach adenocarcinoma (STAD). Conversely, its expression was markedly down-regulated in 10 tumor types: breast cancer (BRCA), colon adenocarcinoma (COAD), glioblastoma (GBM), kidney chromophobe (KICH), kidney renal clear cell carcinoma (KIRC), kidney renal papillary cell carcinoma (KIRP), lung adenocarcinoma (LUAD), lung squamous cell carcinoma (LUSC), pheochromocytoma and paraganglioma (PCPG), and uterine corpus endometrial carcinoma (UCEC) (Fig. 1A). Subsequently, we further compared WWP2 expression between 23 tumor tissues and their paired normal tissues. The results demonstrated that WWP2 expression was significantly elevated in four tumor types (CHOL, LIHC, PRAD, and STAD) compared with their adjacent normal tissues. In contrast, significantly reduced WWP2 expression was observed in eight tumor types: BRCA, COAD, KICH, KIRC, LUAD, LUSC, THCA (thyroid carcinoma), and UCEC (Fig. 1B). Then, a radar plot was generated to display the distribution patterns and statistical differences of WWP2 expression across multiple tumor tissues and adjacent normal tissues (Fig. 1C).

We conducted the prognostic analysis by integrating WWP2 mRNA expression levels with clinical data from the TCGA-LIHC cohort. The survival analysis revealed shorter overall survival in patients with high WWP2 expression compared to those with low expression (HR = 1.69, 95% CI: 1.17–2.45, *P* < 0.05; Fig. 1D). This prognostic disadvantage was also observed in progression-free interval, where the high-expression group showed reduced PFI relative to the low-expression group (HR = 1.39, 95% CI: 1.01–1.92, *P* < 0.05; Fig. 1E). To clinically validate WWP2 expression patterns, we analyzed 30 paired tumor and adjacent normal tissues from HCC patients. Western blot and immunohistochemical analysis revealed significantly elevated WWP2 protein levels in tumor tissues compared with matched normal tissues (Fig. 1F–H). Overall, these consistent findings establish WWP2 as an oncoprotein with significant prognostic value in HCC.

### WWP2 promotes HCC cell proliferation

**Fig. 2:**
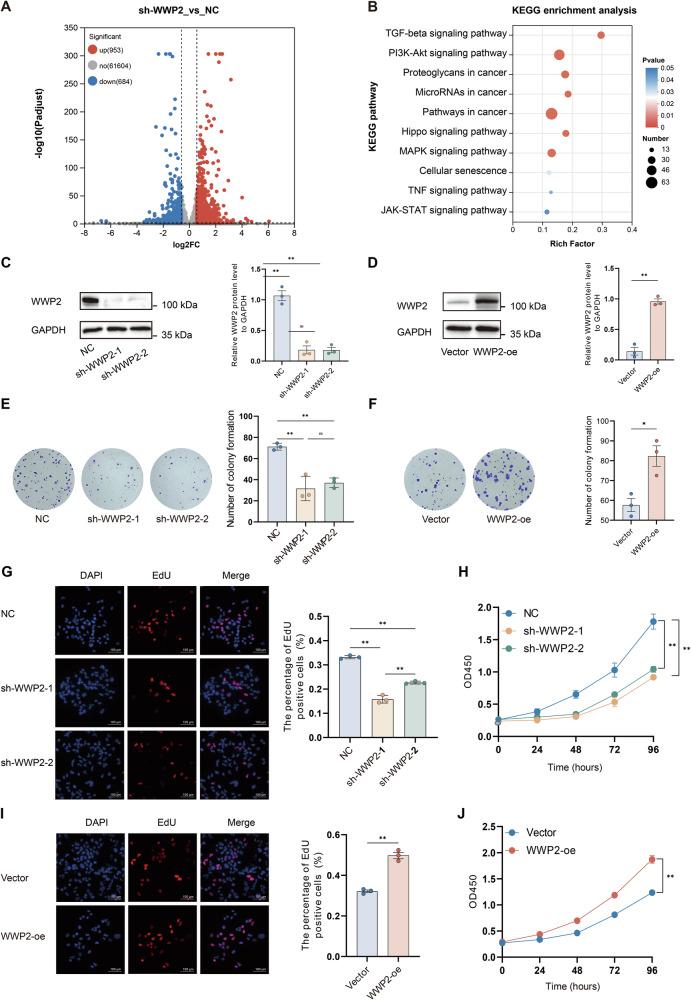
WWP2 regulates the proliferation of HuH-7 cells. **A** Differential gene expression analysis between the WWP2 knockdown group and the NC group. **B** The bubble plot displayed the results of KEGG enrichment analysis for the DEGs. Western blot was used to assess the efficiency of WWP2 knockdown (**C**) or overexpression (**D**) in HuH-7 cells. Colony formation assay assessed the effects of WWP2 knockdown (**E**) or overexpression (**F**) in HuH-7 cells. **G** EdU staining was used to evaluate the effects of WWP2 knockdown on proliferation capacity. **H** CCK-8 assay was performed to evaluate cell proliferation after WWP2 knockdown. **I** EdU staining was used to evaluate the effects of WWP2 overexpression on proliferation capacity. **J** CCK-8 assay was performed to evaluate cell proliferation after WWP2 overexpression. Data presented as mean ± SEM; * *P* < 0.05, **P < 0.01; ns, not significant.

To elucidate the biological functions and molecular mechanisms of WWP2 in HCC, we conducted transcriptome profiling of HuH-7 cells following WWP2 knockdown using specific shRNA, with non-targeting shRNA serving as a negative control. Differential gene expression analysis between the two groups was conducted using the R package DESeq2. With filtering criteria of |Fold Change| ≥ 1.5 and adjusted *p*-value (padj) < 0.05, we identified 1637 differentially expressed genes (DEGs), including 953 upregulated genes and 684 downregulated genes in the WWP2 knockdown group (Fig. 2A). Subsequent KEGG pathway enrichment analysis of these DEGs revealed significant enrichment in the following pathways: TGF-β signaling pathway, PI3K-Akt signaling pathway, Hippo signaling pathway, MAPK signaling pathway, TNF signaling pathway, JAK-STAT signaling pathway, Proteoglycans in cancer, MicroRNAs in cancer, Pathways related to tumorigenesis, Cellular senescence (Fig. 2B).

To explore the functional role of WWP2 in HCC cells, we generated stable WWP2-knockdown and overexpression cell lines via lentiviral transduction, with efficiency confirmed by Western blot (Fig. 2C, D). We then evaluated the effect of WWP2 on cell proliferation using colony formation, EdU staining, and CCK-8 assays. The results indicated that WWP2 knockdown significantly reduced colony formation ability, whereas overexpression enhanced it (Fig. 2E, F). EdU staining revealed a decrease in the proportion of proliferating cells upon WWP2 knockdown and an increase following WWP2 overexpression (Fig. 2G, I). Moreover, CCK-8 assays indicated that WWP2 knockdown suppressed cellular proliferation, while overexpression promoted it (Fig. 2H, J). Collectively, consistent results across multiple proliferation assays demonstrated that WWP2 plays a promotive role in HCC cell proliferation.

### WWP2 modulates cellular senescence by regulating p21 expression in HCC

**Fig. 3:**
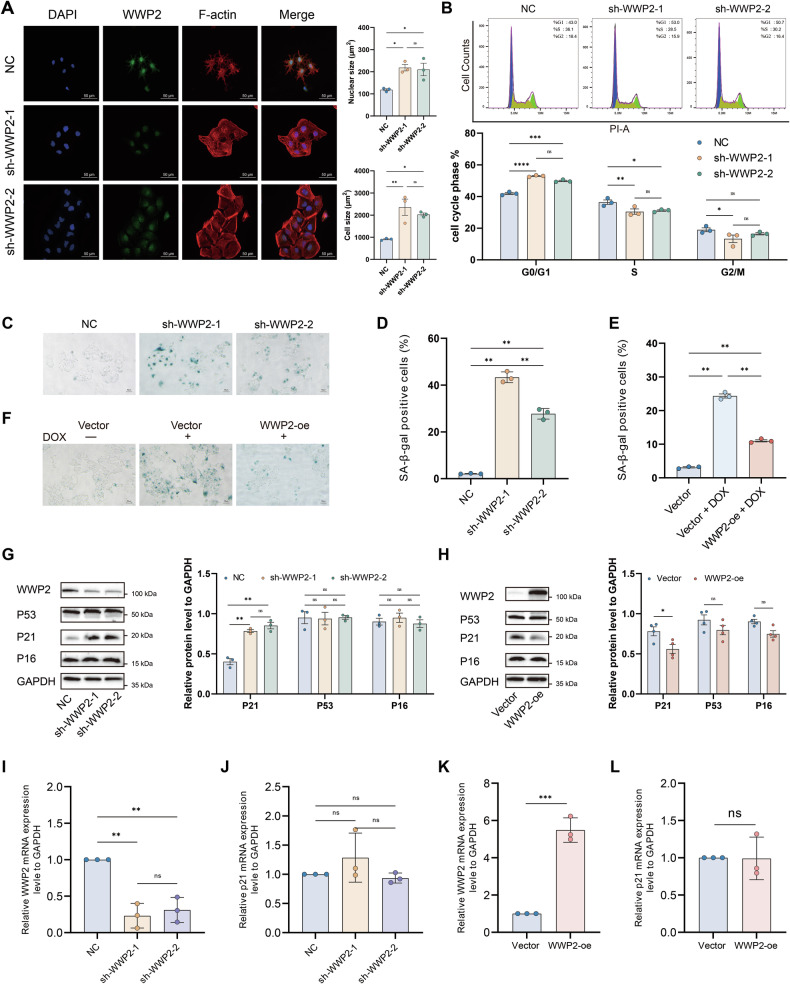
WWP2 regulates p21 expression and modulates senescence in HCC cells. **A** Representative immunofluorescence image of HuH-7 cells stained for F-actin (red), WWP2 (green), and nuclei (DAPI, blue), wherein F-actin and DAPI delineate cellular and nuclear morphology, respectively. **B** Assessment of the cell cycle following WWP2 knockdown using flow cytometry. **C** and **D** Representative images of SA-β-gal staining in HuH-7 cells following WWP2 knockdown, and quantitative analysis of SA-β-gal-positive cells. **E** and **F** Representative images of SA-β-gal staining and quantitative analysis of SA-β-gal–positive cells in WWP2 overexpressing HuH-7 cells with or without DOX treatment. **G** and **H** Western blot and quantitative analysis of p21, p53, and p16 expression in WWP2 knockdown or overexpression HuH-7 cells. **I** and **J** RT-qPCR analysis of WWP2 knockdown efficiency and p21 mRNA expression in HuH-7 cells. **K** and **L** RT-qPCR analysis of WWP2 overexpression efficiency and p21 mRNA expression in HuH-7 cells. Data presented as mean ± SEM; * *P* < 0.05, **P < 0.01, ***P < 0.001, ****P < 0.0001; ns, not significant.

Interestingly, we observed that knockdown of WWP2 induced striking alterations in cellular morphology, notably enlarged nuclei and increased cell size (Fig. 3A). Noteworthily, increased cell volume along with enlarged or multiple nuclei are well-established morphological features of senescent cells [30]. Previously, Wwp2 was identified as a midbrain-specific longevity-associated gene in mice [31]. Furthermore, KEGG pathway enrichment analysis suggested that WWP2 is potentially involved in the cellular senescence pathway. Based on these findings, we further investigated the role of WWP2 in regulating cellular senescence in HCC cells. Cellular senescence is defined as a state of stable cell-cycle arrest triggered by diverse intrinsic and extrinsic stressors [5]. Therefore, we initially examined the impact of WWP2 knockdown on cell cycle progression. The results revealed an induced G1/S phase arrest in HuH-7 cells (Fig. 3B), further supporting WWP2’s role in regulating cellular senescence.

Cellular senescence is also characterized by significantly elevated SA-β-gal activity, which serves as the most widely used biomarker for senescence identification [32]. As anticipated, we observed a marked increase in SA-β-gal-positive cells upon WWP2 depletion in HuH-7 cells (Fig. 3C, D). Extensive evidence demonstrates that doxorubicin (DOX), as a canonical DNA-damaging chemotherapeutic agent, induces genomic instability and triggers cellular senescence [33]. Then, we determined the half-maximal inhibitory concentration (IC₅₀) of DOX in both HuH-7 and HCCLM3 cells and confirmed its senescence-inducing effects (Supplementary Fig. 1A–C). In HuH-7 cells treated with 0.5 μM DOX for 48 h, SA-β-gal staining demonstrated that WWP2 overexpression effectively countered the DOX-induced increase in senescent cells (Fig. 3E, F).

A hallmark of cellular senescence is the robust upregulation of cell cycle inhibitors, including p16, p21, and the primary regulator p53 [7]. We next examined the effect of WWP2 on these proteins in HuH-7 cells. WWP2 knockdown markedly increased p21 expression, but did not alter p53 or p16 levels (Fig. 3G). Conversely, WWP2 overexpression significantly decreased p21, with no notable effect on p53 or p16 (Fig. 3H). These results suggest that WWP2 specifically regulates p21 rather than broadly affecting core senescence-related pathways. Furthermore, RT-qPCR analysis revealed that neither WWP2 knockdown nor overexpression led to significant changes in p21 mRNA expression (Fig. 3I–L), suggesting that the regulation of p21 mediated by WWP2 may occur at the post-translational level.

These results suggest that WWP2 potentially regulates HCC cell senescence by modulating p21 protein levels. This finding prompted us to next investigate the regulatory role of WWP2 in p21 expression and to elucidate the underlying molecular mechanisms.

### WWP2 promotes proteasomal degradation of p21 via K48-linked polyubiquitination

**Fig. 4:**
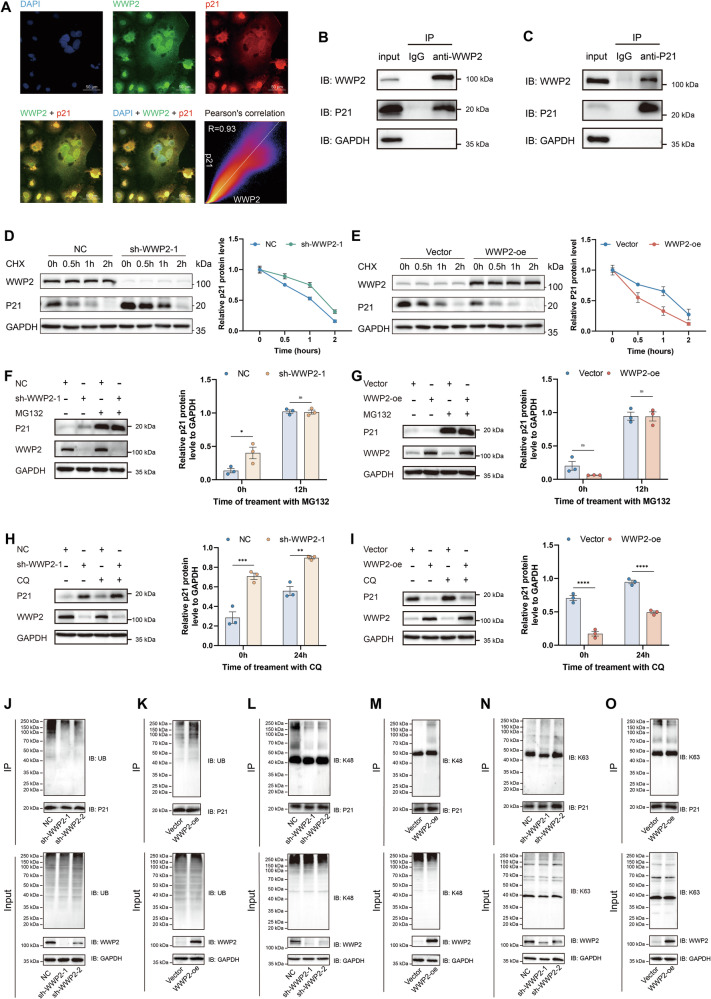
WWP2 regulates p21 stability through ubiquitination. **A** Immunofluorescence analysis of p21 and WWP2 co-localization in HuH-7 cells, with quantification performed using ImageJ’s Coloc2 plugin. **B** Co-IP analysis of the endogenous WWP2-p21 interaction in HuH-7 cells: WWP2 immunoprecipitation followed by p21 immunodetection. **C** Co-IP analysis of the endogenous WWP2-p21 interaction in HuH-7 cells: p21 immunoprecipitation followed by WWP2 immunodetection. **D** and **E** Western blot analysis and protein decay curve of p21 in WWP2 knockdown or overexpressing HuH-7 cells treated with CHX for the indicated times. **F** and **G** Western blot analysis and statistical quantification of p21 protein levels in WWP2 knockdown or overexpressing HuH-7 cells treated with MG132 for 12 hours. **H** and **I** Western blot analysis and statistical quantification of p21 protein levels in WWP2 knockdown or overexpressing HuH-7 cells treated with chloroquine (CQ) for 24 h. **J** and **K** Assessment of p21 ubiquitination in WWP2 knockdown or overexpressing HuH-7 cells. Cells were treated with MG132 for 6 hours prior to analysis. **L** and **M** Analysis of K48-linked polyubiquitination on p21 in WWP2 knockdown or overexpressing HuH-7 cells. **N** and **O** Analysis of K63-linked polyubiquitination on p21 in WWP2 knockdown or overexpressing HuH-7 cells. Data presented as mean ± SEM; *P < 0.05, **P < 0.01, ***P < 0.001, ****P < 0.0001; ns, not significant.

Initially, we examined the subcellular localization of WWP2 and p21 using double immunofluorescence staining. The results showed significant subcellular colocalization of p21 and WWP2 in HuH-7 cells (*R* = 0.93) (Fig. 4A). Then, the reciprocal co-IP experiments conclusively demonstrated the interplay between WWP2 and p21 in HuH-7 cells (Fig. 4B, C), providing mechanistic insight into how WWP2 governs cellular senescence through p21 regulation.

As an E3 ubiquitin ligase, WWP2 catalyzes substrate ubiquitination to modulate protein stability [28]. We then examined the effect of WWP2 on p21 protein stability. After treatment with CHX (100 μg/mL) to block protein synthesis, we determined that WWP2 knockdown prolonged the half-life of p21 (Fig. 4D), whereas WWP2 overexpression shortened it (Fig. 4E), demonstrating that WWP2 promotes the degradation pathway of p21. It is well-known that eukaryotes primarily employ two major protein degradation systems for regulating protein stability: the 26S proteasome and the lysosomal degradation pathway [34]. To investigate the homeostatic regulation mechanism of p21 protein in response to WWP2 expression, we treated HuH-7 cells with the proteasome inhibitor MG132 or the lysosomal inhibitor chloroquine (CQ). Indeed, treatment with MG132 (10 μM, 12 h) rescued the decrease in p21 protein levels induced by WWP2 overexpression and likewise reversed the p21 upregulation resulting from WWP2 knockdown (Fig. 4F, G). In contrast, treatment with CQ (20 μM, 24 h) failed to reverse the decrease in p21 caused by WWP2 overexpression and likewise failed to restore its increase following WWP2 knockdown (Fig. 4H, I). These findings indicated that WWP2 overexpression destabilizes p21 via the proteasome pathway, but not via the lysosomal pathway.

Next, to determine whether WWP2 mediates p21 degradation via polyubiquitination, we assessed p21 ubiquitination by immunoprecipitation in HuH-7 cells following pretreatment with MG132 (10 μM for 6 h). Accordingly, WWP2 knockdown led to reduced p21 polyubiquitination (Fig. 4J). Conversely, WWP2 overexpression promoted robust polyubiquitination of p21 (Fig. 4K). Previous studies have established that WWP2 modifies its substrates via both K48- and K63-linked ubiquitin chains [28]. Therefore, to determine the type of polyubiquitin chain catalyzed by WWP2 in the regulation of p21, we performed endogenous immunoprecipitation in HuH-7 cells. Following treatment with MG132 (10 μM, 6 h), cell lysates were immunoprecipitated with an anti-p21 antibody and subsequently immunoblotted with antibodies specific for K48- and K63-linked ubiquitin chains. The results showed that WWP2 knockdown reduced K48-linked polyubiquitination of p21 (Fig. 4L), whereas WWP2 overexpression enhanced it (Fig. 4M). However, the presence or absence of WWP2 had no apparent effect on the K63-linked polyubiquitination of p21 (Fig. 4N, O).

Taken together, these results identify WWP2 as a novel E3 ubiquitin ligase for p21 that mediates its K48-linked polyubiquitination and thereby promotes proteasomal degradation.

### CMTM6 interacts with WWP2 and regulates p21 protein expression

**Fig. 5:**
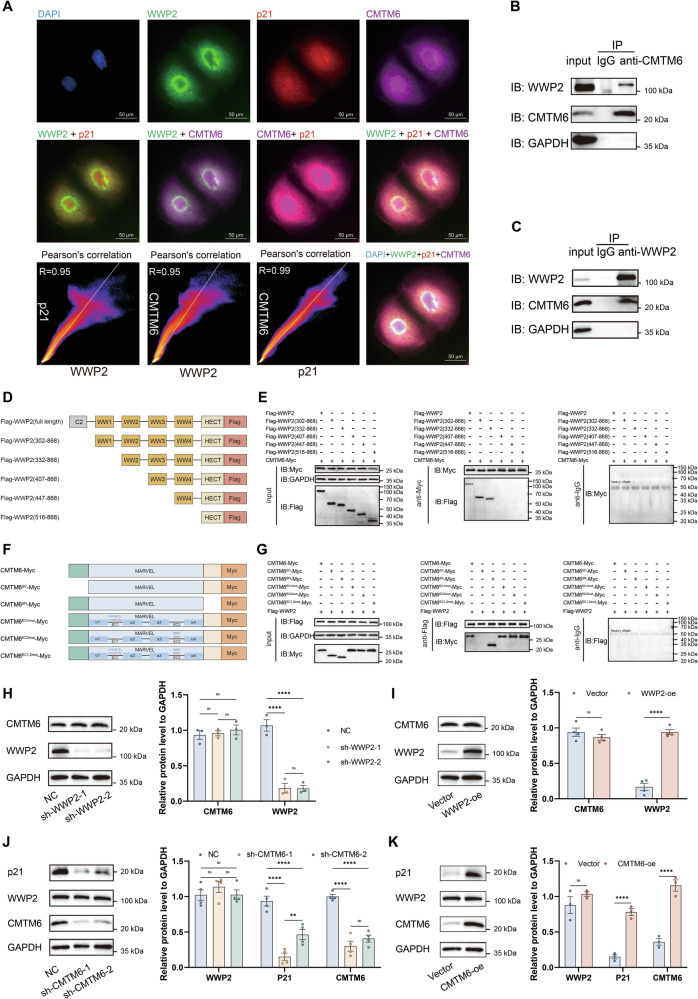
CMTM6 interacts with WWP2 and regulates p21 expression in HCC cells. **A** Immunofluorescence analysis of CMTM6, p21 and WWP2 co-localization in HuH-7 cells, with quantification using ImageJ’s Coloc2 plugin to calculate Pearson correlation coefficient (*R*) values. **B** Co-IP analysis of endogenous WWP2-CMTM6 interaction in HuH-7 cells: CMTM6 immunoprecipitation followed by WWP2 immunodetection. **C** Co-IP analysis of endogenous WWP2-CMTM6 interaction in HuH-7 cells: WWP2 immunoprecipitation followed by CMTM6 immunodetection. **D** Schematic diagram of truncated WWP2 fragments designed for interaction domain mapping. **E** Co-IP to confirm the binding domain of WWP2 for CMTM6. **F** Based on the AlphaFold-predicted structure of human CMTM6, which consists of a transmembrane (TM) MARVEL domain, two extracellular (EC) loops, and intracellular N′ and C′ tails, a series of protein fragments and mutations were designed for domain mapping. Amino acid substitutions (red substituted blue) were introduced into the EC1 and EC2 loops of CMTM6 based on sequence homology with murine CMTM6. **G** Co-IP to confirm the binding domain of CMTM6 for WWP2. **H** and **I** Western blot analysis of CMTM6 expression in HuH-7 cells following WWP2 knockdown or overexpression. **J** and **K** Western blot analysis of p21 and WWP2 expression in HuH-7 cells following CMTM6 knockdown or overexpression. Data presented as mean ± SEM; **P < 0.01, ****P < 0.0001; ns, not significant.

Previous studies have established that CMTM6 physically interacts with p21, stabilizing the protein by preventing its ubiquitin-mediated degradation, which subsequently induces cell cycle arrest and inhibits HCC cell proliferation [15]. To investigate the potential role of CMTM6 in WWP2-mediated p21 regulation, we first examined the subcellular localization of these proteins via triple-label immunofluorescence staining in HuH-7 cells. The results demonstrated strong co-localization between all three pairs, with high Pearson’s correlation coefficients: CMTM6/WWP2 (*R* = 0.95), CMTM6/p21 (*R* = 0.99), and p21/WWP2 (R = 0.95) (Fig. 5A). Furthermore, the reciprocal co-IP assays conclusively demonstrated the interplay between WWP2 and CMTM6 in HuH-7 cells (Fig. 5B, C).

Subsequently, to identify the CMTM6-binding domains of WWP2, we generated a series of truncated WWP2 constructs for interaction mapping (Fig. 5D). We found that ablation of the WW2 domain resulted in a complete loss of interaction, indicating that the C2 domain, WW1, WW3, and WW4 domains, as well as the HECT domain, are dispensable for binding, while the WW2 domain is essential for the interaction with CMTM6 (Fig. 5E). Based on previous studies showing that CMTM6 is primarily composed of a large, conserved MARVEL domain with four transmembrane helices and two extracellular loops (EC1 and EC2), along with structurally uncharacterized intracellular N- and C-terminal tails [35]. Considering that its extracellular loops are required for direct binding to both CD58 and PD-L1 [35], we generated a series of CMTM6 truncation or mutant constructs for interaction domain mapping (Fig. 5F). According to the domain mapping results, WWP2 directly binds to CMTM6, likely via its C-terminal intracellular tail (Fig. 5G).

Thereafter, we examined the regulatory relationship between CMTM6 and WWP2. Notably, neither WWP2 knockdown nor overexpression affected CMTM6 protein expression levels (Fig. 5H, I). Similarly, neither CMTM6 knockdown nor overexpression altered WWP2 expression. However, consistent with prior findings, p21 expression was significantly reduced upon CMTM6 knockdown and elevated following CMTM6 overexpression (Fig. 5J, K). Overall, the direct interaction between WWP2 and CMTM6 serves as a mechanism to modulate p21 protein abundance independent of the regulation of either WWP2 or CMTM6 expression.

### CMTM6 antagonizes WWP2-mediated regulation of HCC cell proliferation and cellular senescence

**Fig. 6:**
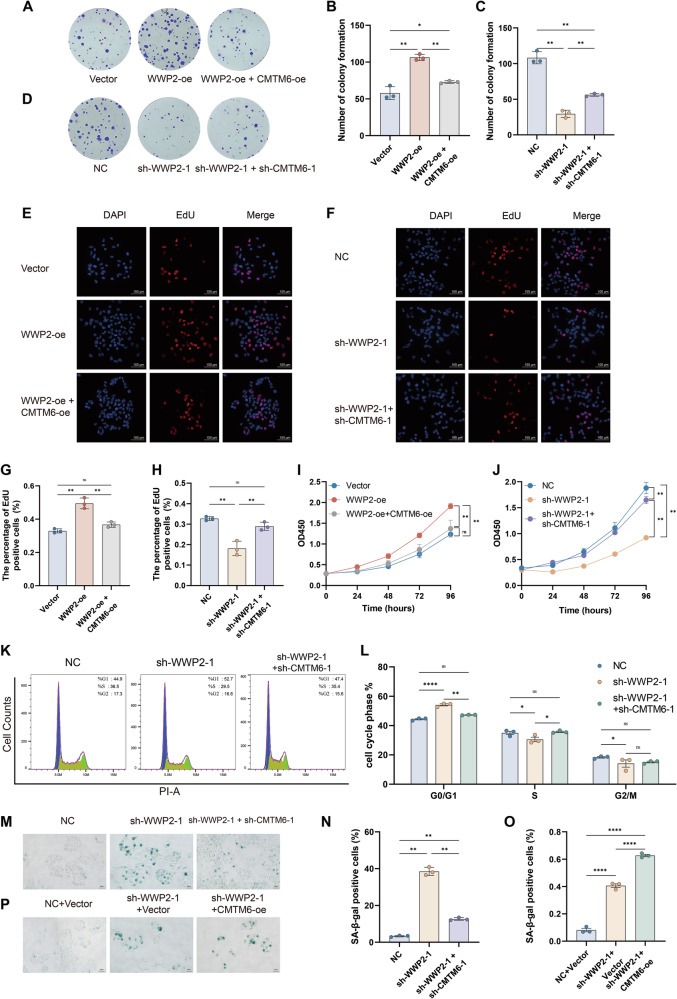
WWP2 and CMTM6 coordinated regulation of proliferation and cellular senescence in HCC cells. **A** and **B** Colony formation assay was used to analyze the proliferative capacity of HuH-7 cells following WWP2 overexpression alone or combined with CMTM6 overexpression. **C** and **D** Colony formation assay was used to analyze the proliferative capacity of HuH-7 cells following WWP2 knockdown alone or combined with CMTM6 knockdown. **E** and **G** EdU staining illustrated the proliferative dynamics of HuH-7 cells following WWP2 overexpression alone or combined with CMTM6 overexpression. **F** and **H** EdU staining illustrated the proliferative dynamics of HuH-7 cells following WWP2 knockdown alone or combined with CMTM6 knockdown. **I** The CCK-8 assay was used to measure the proliferation rates of HuH-7 cells following WWP2 overexpression alone or combined with CMTM6 overexpression. **J** The CCK-8 assay was used to measure the proliferation rates of HuH-7 cells following WWP2 knockdown alone or combined with CMTM6 knockdown. **K** and **L** Flow cytometry was performed to assess cell cycle distribution following WWP2 knockdown alone or combined with CMTM6 knockdown. **M** and **N** SA-β-gal staining was employed to detect cellular senescence in HuH-7 cells following WWP2 knockdown alone or combined with CMTM6 knockdown. **O** and **P** SA-β-gal staining was employed to detect cellular senescence of HuH-7 cells following WWP2 knockdown or WWP2-konckdown with CMTM6 overexpression. Data presented as mean ± SEM; *P < 0.05, **P < 0.01, ****P < 0.0001; ns, not significant.

To elucidate the functional impact of CMTM6 on WWP2-mediated regulation in HCC, we generated two novel stable HuH-7 cell models: dual-knockdown (sh-WWP2-1+sh-CMTM6-1) and co-overexpression (WWP2-oe+CMTM6-oe) cell lines. The results from colony formation (Fig. 6A–D), EdU staining (Fig. 6E–H), and CCK-8 assays (Fig. 6I, J) consistently demonstrated that the proliferation suppression resulting from WWP2 deficiency was partially reversed by co-knockdown of CMTM6. Conversely, the proliferation enhancement resulting from WWP2 overexpression was sharply attenuated by co-overexpression of CMTM6. Subsequent cell cycle analysis revealed that WWP2 knockdown induced cell cycle arrest, which was partially rescued by concurrent CMTM6 knockdown (Fig. 6K, L). Additionally, SA-β-gal staining revealed that CMTM6 knockdown partially reduced the increase in senescent cells induced by WWP2 knockdown (Fig. 6M, N).

Furthermore, to explore the synergistic function of CMTM6 and WWP2 in HCC progression, we transfected a CMTM6 expression vector into WWP2-knockdown HuH-7 cells to generate a model with concurrent CMTM6 overexpression and WWP2 knockdown (Supplementary Fig. 2A). Functional assays showed that WWP2 knockdown impaired colony formation, cell proliferation, and cell cycle progression. Notably, these inhibitory effects were significantly exacerbated by concurrent CMTM6 overexpression (Supplementary Fig. 2B–D). In addition to proliferation and cell cycle alterations, we also assessed cellular senescence. As expected, the percentage of SA-β-gal-positive cells was further elevated in the combined treatment group compared to WWP2 knockdown alone (Fig. 6O, P).

In summary, these results demonstrate that CMTM6 counteracts WWP2’s function, thereby antagonizing the pro-proliferative and anti-senescent effects mediated by WWP2 in HCC.

### CMTM6 modulates WWP2-mediated p21 ubiquitination

**Fig. 7:**
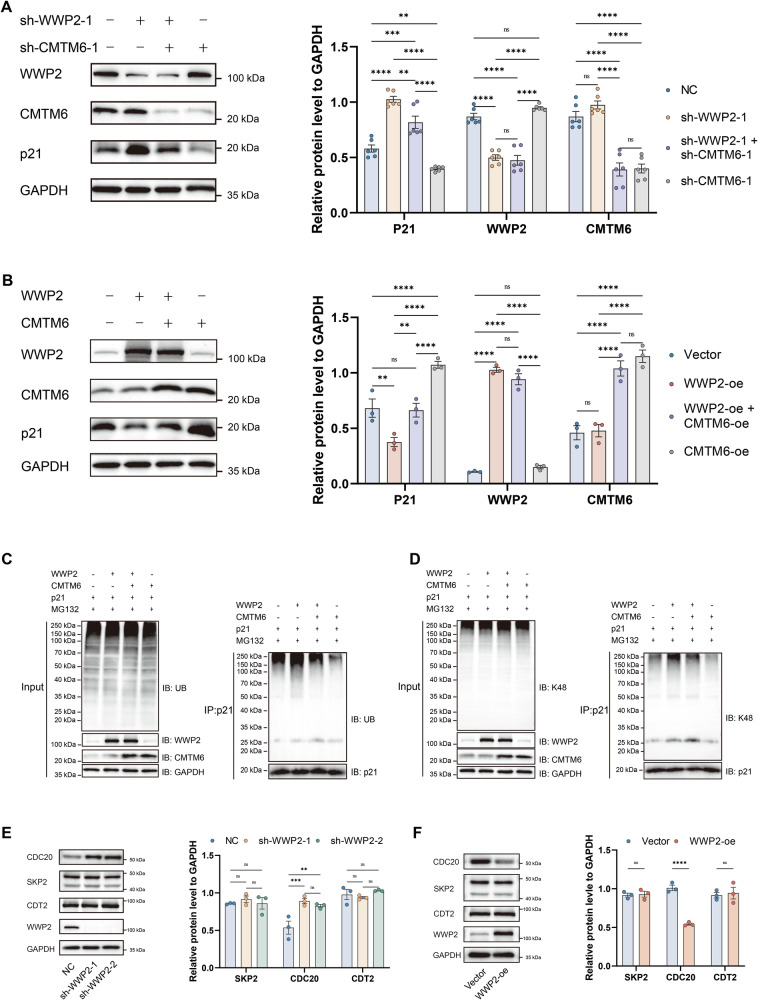
WWP2 and CMTM6 synergistically regulate p21 ubiquitination in HCC cells. **A** Western blot analysis of p21, CMTM6, and WWP2 expression in HuH-7 cells under distinct genetic perturbations: WWP2 knockdown, CMTM6 knockdown, or WWP2 and CMTM6 dual-knockdown. **B** Western blot analysis of p21, CMTM6, and WWP2 expression in HuH-7 cells under distinct genetic perturbations: WWP2 overexpression, CMTM6 overexpression, or WWP2 and CMTM6 dual-overexpression. **C** p21 ubiquitination in p21-overexpressing HuH-7 cells co-transfected with empty vector, WWP2, CMTM6, or both WWP2 and CMTM6. **D** K48-linked p21 ubiquitination in p21-overexpressing HuH-7 cells co-transfected with empty vector, WWP2, CMTM6, or both WWP2 and CMTM6. **E** and **F** Western blot assessment of CDC20, SKP2 and DTL expression profiles in HuH-7 cells subjected to WWP2 knockdown or overexpression. Data presented as mean ± SEM; **P < 0.01, ***P < 0.001, ****P < 0.0001; ns, not significant.

Subsequently, we investigated the regulatory role of the CMTM6–WWP2 interaction in mediating p21 expression in HuH-7 cells. The results indicated that CMTM6 knockdown partially reversed the upregulation of p21 resulting from WWP2 knockdown (Fig. 7A). Similarly, CMTM6 overexpression partially rescued the suppression of p21 expression induced by WWP2 overexpression (Fig. 7B). Next, we investigated whether CMTM6 affects WWP2-mediated ubiquitination of p21. To this end, HuH-7 cells were transiently co-transfected with a p21 plasmid along with plasmids encoding empty vector (control), WWP2, CMTM6, or both WWP2 and CMTM6. Following MG132 treatment, anti-p21 immunoprecipitation and Western blot analysis revealed that WWP2 overexpression enhanced p21 ubiquitination, an effect partially attenuated by CMTM6 co-overexpression (Fig. 7C). Using a K48-linkage-specific antibody, we further confirmed that WWP2 overexpression increased K48-linked ubiquitination of p21, which could be partially reduced by CMTM6 co-overexpression (Fig. 7D). These findings firmly establish that CMTM6 negatively regulates WWP2-mediated K48-linked ubiquitination of p21.

Previous studies have demonstrated that CMTM6 stabilizes p21 by protecting it from ubiquitin-dependent degradation mediated by the SCF^SKP2^, CRL4^CDT2^, and APC/C^CDC20^ E3 ubiquitin ligase complexes [15]. Therefore, we examined whether WWP2 regulates the expression of these molecules. Interestingly, neither knockdown nor overexpression of WWP2 significantly affected SKP2 or CDT2 levels. However, WWP2 knockdown or overexpression increased or decreased CDC20 expression, respectively (Fig. 7E, F). The results indicate that WWP2 not only directly promotes the degradation of p21 but also indirectly enhances p21 stability by negatively regulating CDC20—an E3 ubiquitin ligase that targets p21—thus forming a complex regulatory network. We propose that the direct degradation of p21 by WWP2 and its indirect stabilization via CDC20 may cooperate within cells to maintain the dynamic equilibrium of p21 protein levels. However, this regulatory model requires further experimental validation.

### WWP2 and CMTM6 regulate HCC tumor growth and senescence in Vivo

**Fig. 8:**
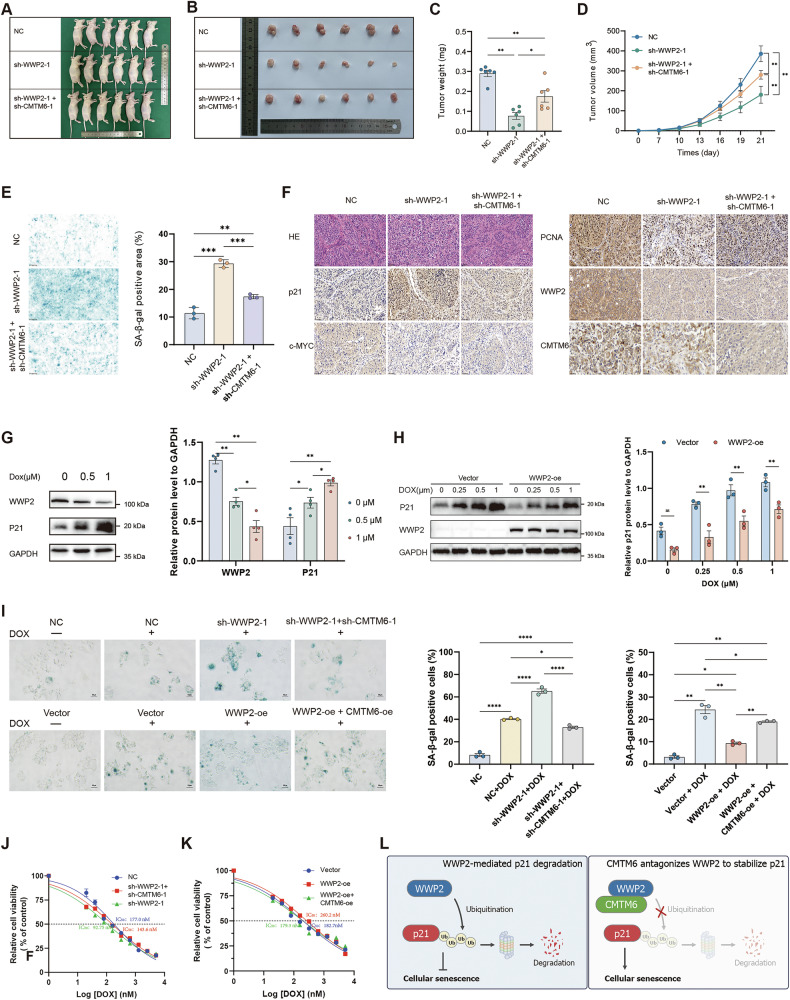
WWP2 depletion suppressed HCC tumor growth in vivo and induced DOX sensitivity by promoting cellular senescence, effects that were antagonized by CMTM6. Images of nude mice with tumors (**A**) and subcutaneous tumors (**B**) in the WWP2-knockdown group, WWP2 and CMTM6 dual-knockdown group, and NC group. Tumor weights (**C**) and tumor volumes (**D**) were measured for each group (*n* = 6 per group). **E** Representative images of SA-β-gal staining performed on frozen sections of subcutaneous tumor tissues and the statistical quantification of the area percentage of positively stained regions. **F** Representative images of H&E staining and immunohistochemical staining for p21, c-MYC, PCNA, WWP2, and CMTM6 expression in subcutaneous tumors. **G** Western blot analysis of p21 expression in HuH-7 cells treated with a gradient concentration series of DOX for 48 h. **H** Western blot analysis of p21 expression in WWP2 overexpression HuH-7 cells treated with a gradient concentration series of DOX for 48 h. **I** SA-β-gal activity and quantification of positive cells in HuH-7 cells grouped by WWP2 and CMTM6 expression status after 48 h DOX induction. **J** Dose–response curves of DOX treatment for 48 h in HuH-7 cells (IC₅₀ values indicated) following WWP2 knockdown alone or WWP2/CMTM6 dual knockdown. **K** Dose–response curves of DOX treatment for 48 h in HuH-7 cells (IC₅₀ values indicated) following WWP2 overexpression alone or WWP2/CMTM6 dual overexpression. **L** The schematic specifically depicts the functional interplay between WWP2 and CMTM6 in mediating p21 protein degradation and cellular senescence induction in HCC cells. Data presented as mean ± SEM; *P < 0.05, **P < 0.01, ***P < 0.001, ****P < 0.0001; ns, not significant.

Based on the in vitro data above, we further investigated the role of WWP2 and CMTM6 via xenograft tumor models in vivo. To establish a xenograft tumor model, nude mice were subcutaneously implanted with HuH-7 cells from the following groups: WWP2 knockdown alone, WWP2/CMTM6 dual knockdown, and negative control. As shown in Fig. 8A and B, the photo of tumor-bearing nude mice and excised subcutaneous tumor tissues is displayed. Tumors from the WWP2-knockdown group exhibited significantly lower weight than those from the NC group, an effect that was partially reversed by concurrent CMTM6 knockdown (Fig. 8C). Similarly, tumor growth curves demonstrated that WWP2 knockdown markedly suppressed tumor growth, which was also rescued by CMTM6 knockdown (Fig. 8D).

To confirm the regulatory role of WWP2 and CMTM6 in HCC cell senescence in vivo, frozen sections of tumor tissues were subjected to SA-β-gal staining. We observed that HCC tissues with WWP2 knockdown exhibit a higher proportion of senescent cells, which was attenuated by concurrent CMTM6 knockdown (Fig. 8E). In addition, tumor tissues were stained with H&E to observe tumor cell morphology, and immunohistochemistry (IHC) was performed to detect the expression level of p21, WWP2, CMTM6, the cell cycle promoter c-MYC, and the proliferation marker PCNA. H&E staining confirmed that the collected specimens consisted of HCC tumor tissues. IHC analysis revealed that WWP2 knockdown reduced the expression of PCNA and c-MYC, while increasing p21 expression. Again, these effects were partially reversed by CMTM6 knockdown (Fig. 8F).

Taken together, WWP2 knockdown suppresses HCC tumor growth and promotes senescence in vivo, and these effects are abrogated by CMTM6 knockdown, confirming our cellular findings.

### WWP2/CMTM6 regulates DOX-induced senescence and chemosensitivity in HCC

DOX is a widely used chemotherapeutic agent in interventional treatments for HCC, particularly in TACE. It exerts antitumor effects primarily by disrupting DNA synthesis and inhibiting topoisomerase II [36]. Additionally, DOX can induce therapy-induced senescence, which influences therapeutic outcomes in HCC [37]. However, the clinical efficacy of DOX is often limited by drug resistance and systemic toxicity. Overcoming these challenges requires the exploration of rational combination therapies. A deeper understanding of the mechanisms underlying DOX-induced senescence may provide strategies to circumvent resistance and improve treatment efficacy.

Initially, we performed western blot analysis to evaluate the expression levels of WWP2 and p21 in HCC cells treated with varying concentrations of DOX. The results demonstrated that in both HuH-7 and HCCLM3 cell lines, WWP2 expression decreased significantly in a dose-dependent manner with increasing DOX concentration, while p21 protein levels were substantially upregulated, demonstrating an inverse correlation with WWP2 (Fig. 8G and Supplementary Fig. 1D). Moreover, overexpression of WWP2 during DOX treatment effectively attenuates DOX-induced p21 accumulation (Fig. 8H and Supplementary Fig. 1E). Additionally, under DOX-induced senescence conditions, WWP2 overexpression reduced the proportion of senescent cells, partially reversed by CMTM6 overexpression; conversely, WWP2 knockdown enhanced DOX-induced senescence, partially rescued by CMTM6 knockdown (Fig. 8I). To investigate the influence of WWP2 and CMTM6 on DOX sensitivity, cells were treated with indicated concentrations of DOX for 72 h. Cell viability assays revealed that WWP2 overexpression decreased the sensitivity of HCC cells to DOX, whereas WWP2 knockdown increased sensitivity, as indicated by changes in the half-maximal inhibitory concentration (IC₅₀). Notably, modulation of CMTM6 expression counteracted the effects of WWP2 on DOX sensitivity (Fig. 8J, K).

In summary, our study identifies the WWP2/CMTM6 axis as a key regulator of DOX-induced senescence and chemoresistance in HCC. Specifically, WWP2 downregulation promotes p21-mediated senescence and enhances chemosensitivity, while CMTM6 counteracts these effects. Targeting the WWP2/CMTM6 interaction represents a promising therapeutic strategy for overcoming DOX resistance in HCC.

## Discussion

Cellular senescence is a crucial stress response mechanism designed to eliminate unwanted, damaged, or abnormal cells [38]. This process involves stable proliferative arrest and the expression of the SASP [39]. Through SASP, senescent cells recruit immune cells to facilitate immune clearance, thereby restoring tissue homeostasis [10]. Given the growth arrest and immune surveillance-promoting properties of senescent cells, these features provide a theoretical foundation for developing senescence-inducing agents in malignant tumor therapy. For example, AZD7121, an orally active vascular endothelial growth factor receptor-2 (VEGFR-2) tyrosine kinase inhibitor, suppresses tumor growth by inducing cellular senescence and exhibits limited toxicity in phase II clinical trials of advanced HCC patients [40]. This study demonstrates that knockdown of WWP2 inhibited proliferation and induced cellular senescence in HCC cells. In vivo experiments demonstrated that WWP2 knockdown led to increased tumor cell senescence and suppression of tumor growth. These findings align with previous studies, indicating that inducing cellular senescence is an effective therapeutic strategy for HCC. Targeting WWP2-mediated senescence induction may offer a novel potential therapeutic approach for HCC.

Cells primarily enter senescence via critical regulatory pathways: the p53/p21 pathway and the p16/Rb pathway [41]. While both p21 and p16 mediate cell cycle arrest during senescence induction, they exhibit fundamental mechanistic divergences in regulatory pathways. The p16 protein competitively binds to CDK4/6 with cyclin D, inhibiting RB protein phosphorylation and preventing its degradation, thereby blocking the G1/S transition [42]. In contrast, p21 inhibits the cell cycle through dual mechanisms: during the G1 phase, p21 suppresses cyclin D-CDK4/6 complex activity to facilitate DNA damage repair; at the G1/S checkpoint, p21 primarily induces G1 arrest by inhibiting cyclin E-CDK2 and cyclin A-CDK2 activity. This inhibition leads to hypophosphorylated pRB, preventing the release of E2F transcription factors and subsequent S-phase entry. Additionally, p21-mediated suppression of cyclin B-CDK1 activity induces G2 and G2/M phase arrest [14]. Beyond differences in cell cycle arrest mechanisms, p16 and p21 also diverge in senescence-associated subpopulations, stress regulation, secretory phenotypes, and lifespan impacts [14]. Thus, clarifying the regulatory mechanisms of distinct senescence pathways is critical for precise therapeutic interventions. In this study, we found that p53 and p16 protein levels remained unchanged upon WWP2 knockdown or overexpression, whereas p21 expression was negatively regulated by WWP2. These results suggested that WWP2 primarily modulates the senescence phenotype in HCC cells via the p21 pathway.

During senescence regulation, p21 controls the cell cycle through p53-dependent and p53-independent pathways. In the p53-dependent pathway, DNA damage or oxidative stress activates p53, which binds specific response elements in the p21 promoter to induce p21 expression and cell cycle arrest [43]. In p53-independent pathways, p21 expression can be regulated by upstream factors such as c-Myc [44]. This study revealed subcellular co-localization of p21 and WWP2 in HCC cells, and co-immunoprecipitation confirmed their direct interaction. Furthermore, WWP2 regulates p21 protein stability, thereby controlling HCC cell senescence. These results demonstrate that WWP2-mediated p21 regulation operates independently of p53.

The ubiquitin–proteasome system is the primary protein degradation system in eukaryotic cells, responsible for degrading over 80% of intracellular proteins [45]. Among ubiquitin chain types, K48- and K63-linked chains are the most extensively studied. K48-linked polyubiquitination primarily targets substrates for proteasomal degradation, whereas K63-linked chains play non-proteolytic roles in inflammation and DNA damage responses [46]. Ubiquitination critically regulates p21 expression in cancer cells. For instance, FBXO22 upregulation in HCC promotes tumor growth and proliferation by mediating p21 ubiquitination and degradation [16]. Current evidence suggests that targeting genes upstream of the p21 cascade to induce its expression may represent an effective senescence-based therapeutic strategy. For example, the senolytic drug ABT-737 (a Bcl-2 inhibitor) eliminates prolonged p21 expression and promotes liver regeneration [47]. Here, we demonstrate that the E3 ubiquitin ligase WWP2 inhibits cellular senescence in HCC through binding to p21, mediating its K48-linked polyubiquitination, and thereby promoting proteasomal degradation. As a key upstream regulator of p21, WWP2 represents a potential therapeutic target for inducing senescence via the p21 pathway, thereby significantly suppressing HCC growth.

Notably, a previous study demonstrated that WWP2 exhibits dual roles in HCC, acting as both an oncogene and a tumor suppressor. Pro-tumorigenically, WWP2 promotes HCC progression by targeting the PTEN/PI3K/AKT pathway and upregulating CXCR3/CCR5 to enhance proliferation, adhesion, invasion, and migration while inhibiting apoptosis [48, 49]. Conversely, WWP2 suppresses tumor-initiating cell activity by degrading the pluripotency factor OCT4, delaying HCC progression [50]. Our in vitro and in vivo experiments confirmed that WWP2 knockdown promotes cellular senescence and suppresses HCC proliferation, aligning with its tumor-promoting role. These findings highlight WWP2’s context-dependent regulatory functions, where its pro- or anti-tumor effects depend on specific substrate interactions. Future studies must delineate WWP2’s substrate spectrum and its contributions to tumorigenesis. Therapeutic strategies targeting WWP2 may require substrate-specific approaches: inhibiting oncogenic substrates while activating tumor-suppressive ones.

CMTM6 is a member of the chemokine-like factor MARVEL transmembrane domain family [51]. Recently, it has garnered significant attention for its crucial role in regulating the membrane expression stability of programmed cell death ligand 1 (PD-L1) [52]. Mechanistically, CMTM6 prevents lysosomal degradation of PD-L1 by inhibiting STUB1-mediated ubiquitination [53]. Furthermore, CMTM6 stabilizes PD-L1 expression on antigen-presenting cells and enhances PD-1-mediated inhibitory signaling in T cells, thereby triggering T cell suppression and exhaustion [54]. Subsequent studies revealed that in HCC cells, CMTM6 interacts with p21 and stabilizes its protein expression level, leading to blockade of G1/S phase transition, ultimately suppressing HCC cell proliferation [15]. In the present study, through CMTM6 knockdown and overexpression experiments in HCC cells, the results showed a positive regulatory effect of CMTM6 on p21 expression. Consistent with prior reports, we confirmed CMTM6’s positive regulation of p21 in HCC cells.

Intriguingly, our study identified a novel protein-protein interaction between CMTM6 and WWP2 that significantly modulates WWP2’s ubiquitination regulatory function toward p21. Mechanistically, CMTM6 expression inhibits WWP2-mediated p21 ubiquitination, potentially through suppressing the E3 ligase enzymatic activity of WWP2. Previous studies have established that HECT-type E3 ligases typically maintain autoinhibited conformations through intramolecular or intermolecular interactions that lock the HECT domain in an inactive state, preventing hyperactive auto-ubiquitination or excessive substrate ubiquitination [55]. As a HECT family member, WWP2 not only exhibits this multilayered autoinhibition mechanism but also critically depends on intramolecular C2-HECT domain interactions to sustain its inactive conformation [28]. For instance, cadherin 1 enhances WWP2’s autoinhibition by directly engaging both its C2 and HECT domains, thereby reinforcing intramolecular interactions and suppressing WWP2’s E3 ligase activity [56]. In our study, domain mapping analysis revealed that the WW2 domain of WWP2 is essential for binding to CMTM6. Furthermore, mapping of CMTM6 demonstrated that WWP2 directly binds to the C-terminal intracellular tail of CMTM6. Such structural elucidation will directly inform the rational design of compounds targeting these interfaces, thereby accelerating selective drug discovery. Collectively, these findings establish CMTM6 as a pivotal modulator of the WWP2–p21 regulatory axis in HCC.

However, several limitations warrant consideration. First, the structural interactions and precise binding mechanisms among WWP2 and p21 require further elucidation. Second, systematic therapeutic evaluation of WWP2 as a druggable target remains to be explored. Notably, the discovery of NSC288387, a small-molecule inhibitor of WWP2, established the pharmacological potential of targeting this ubiquitin ligase [57]. Building on these findings, our future research will focus on: (1) identifying ubiquitin-acceptor lysine residues within the WWP2–p21 complex using complementary structural biology approaches; and (2) systematically evaluating WWP2-targeted inhibitors in patient-derived xenograft models and organoid systems. These efforts aim to develop safe therapeutic agents targeting the WWP2–p21 axis, ultimately translating mechanistic discoveries into clinical applications for HCC management.

### Conclusion

In summary, this study reveals a novel regulatory mechanism of HCC cellular senescence, as illustrated in Fig. 8L. We demonstrate that WWP2 interacts with p21 to mediate its K48-linked ubiquitination and proteasomal degradation, thereby evading senescence to promote HCC progression. Furthermore, we identify CMTM6 as a crucial regulator of the WWP2–p21 axis. Specifically, CMTM6 directly interacts with WWP2 and antagonizes WWP2-mediated ubiquitination of p21, consequently accelerating cellular senescence and suppressing HCC progression.

## Supplementary information

Supplementary Legends

Table S1

Supplementary Figures

Original Western Blots

Reproducibility Checklist

## Data Availability

Data are available in a public, open-access repository. Data are available upon reasonable request.
