# Supplementary material for: WWP2-induced inhibition of hepatocellular carcinoma cellular senescence via the ubiquitination and degradation of p21
Source: Cell Death Dis. 2025 Dec 12;17(1):96. doi: 10.1038/s41419-025-08318-0 (PMC12830804; doi:10.1038/s41419-025-08318-0)
Supplement: Supplementary file 1 — Supplementary Legends [file 41419_2025_8318_MOESM1_ESM.docx]

**Supplementary information**

Table S1. The hairpin target sequences of WWP2 and CMTM6 genes.

Supplementary Figures.

Original Western Blots.

Reproducibility Checklist.
