# Supplementary material for: WWP2-induced inhibition of hepatocellular carcinoma cellular senescence via the ubiquitination and degradation of p21
Source: Cell Death Dis. 2025 Dec 12;17(1):96. doi: 10.1038/s41419-025-08318-0 (PMC12830804; doi:10.1038/s41419-025-08318-0)
Supplement: Supplementary file 2 — Table S1 [file 41419_2025_8318_MOESM2_ESM.docx]

Table S1. The hairpin target sequences of WWP2 and CMTM6 genes.

| Gene | Target sequence |
| --- | --- |
| sh-WWP2-1 | 5-CCCAAGGTGCATAATCGTCAA-3 |
| sh-WWP2-2 | 5-CTCACCTACTTTCGCTTTATA-3 |
| sh-CMTM6-1 | 5-CCCAAGACAGTGAAAGTAATT-3 |
| sh-CMTM6-2 | 5-TGGAGAACGGAGCGGTGTACA-3 |
