# Supplementary material for: WWP2-induced inhibition of hepatocellular carcinoma cellular senescence via the ubiquitination and degradation of p21
Source: Cell Death Dis. 2025 Dec 12;17(1):96. doi: 10.1038/s41419-025-08318-0 (PMC12830804; doi:10.1038/s41419-025-08318-0)
Supplement: Supplementary file 3 — Supplementary Figures [file 41419_2025_8318_MOESM3_ESM.pdf]

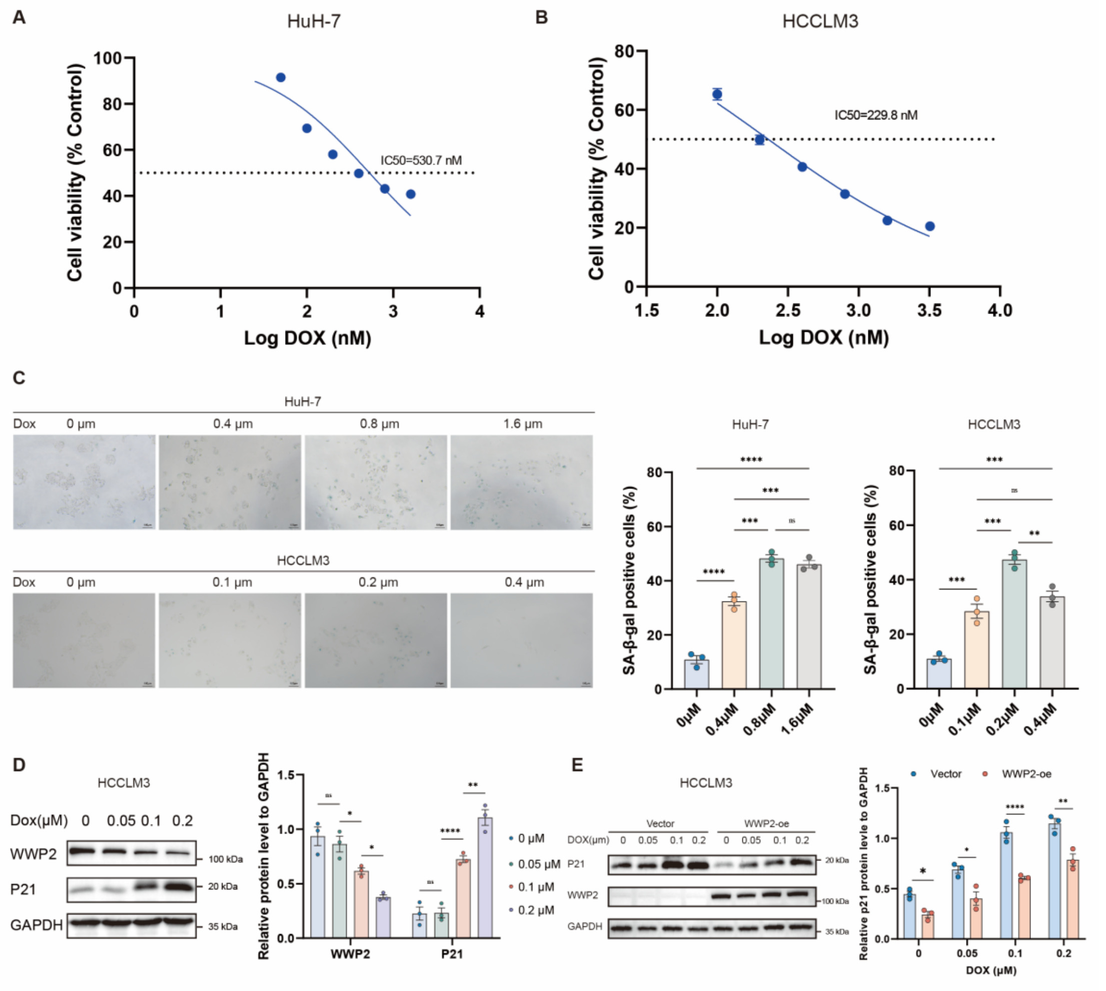

Supplementary Fig. 1 DOX induces cellular senescence in HCC cells. **A** Dose-response curves of DOX treatment for 48 hours in HuH-7 cells (IC_{50} values indicated). **B** Dose-response curves of DOX treatment for 72 hours in HCCLM3 cells (IC_{50} values indicated). **C** Assessment of SA-β-gal activity in HuH-7 and HCCLM3 cells following DOX induction for 48h and 72h, with quantitative analysis of SA-β-gal positive cells. **D** Western blot analysis of p21 and WWP2 expression in HCCLM3 cells treated with increasing concentrations of DOX for 72 hours. **E** Western blot analysis of p21 expression in WWP2-overexpressing HCCLM3 cells treated with increasing concentrations of DOX for 72 hours. Data presented as mean ± SEM; * $P < 0.05$, ** $P < 0.01$, *** $P < 0.001$, **** $P < 0.0001$; ns, not significant.

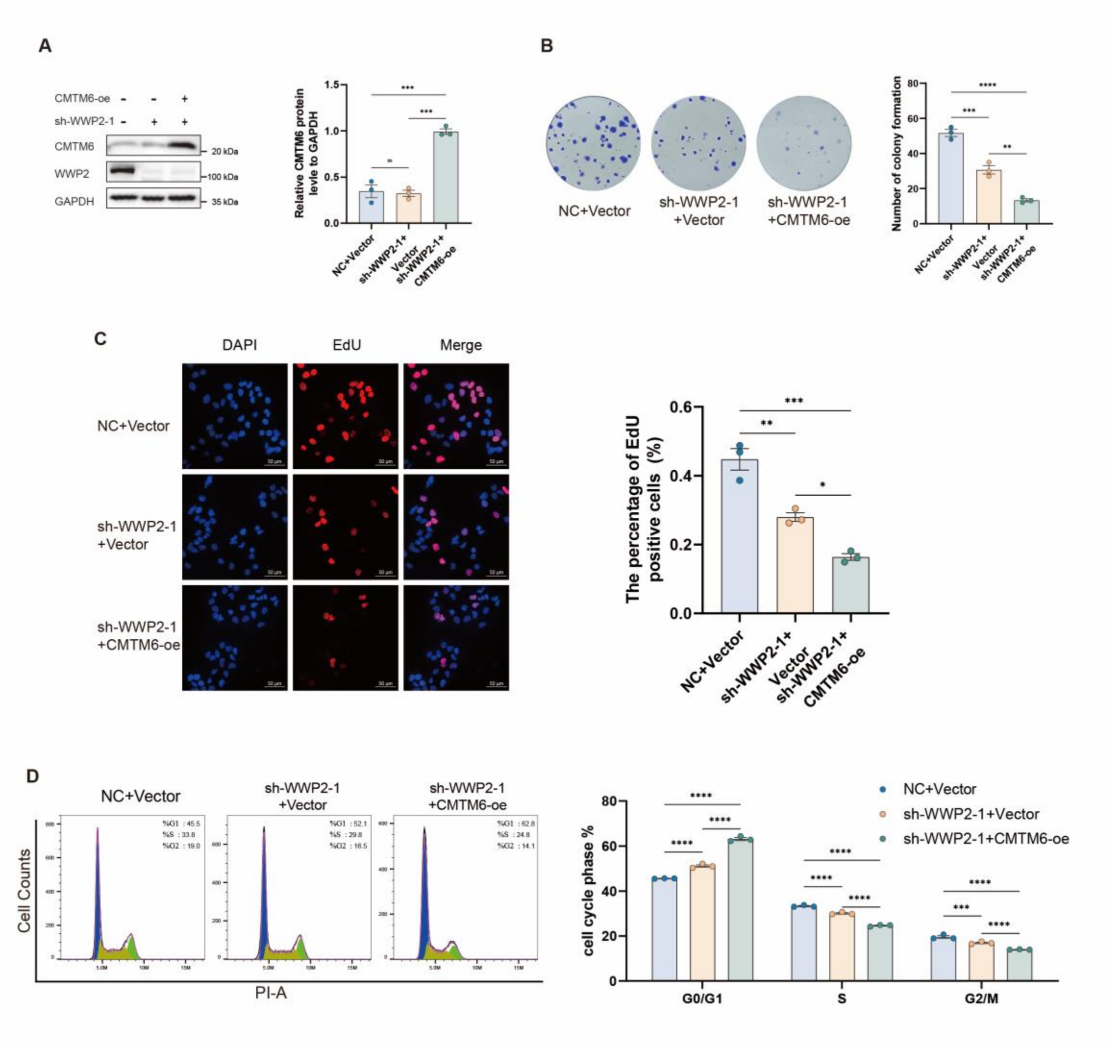

Supplementary Fig. 2 WWP2 knockdown and CMTM6 overexpression synergistically inhibited the proliferation of HCC cells. **A** Western blot to assess the efficiency of CMTM6 overexpression and WWP2 knockdown. **B** Colony formation assay assessed the effects of WWP2 knockdown alone or with CMTM6 overexpression. **C** EdU staining to evaluate the effects of WWP2 knockdown alone or combined with CMTM6 overexpression on cell proliferation. **D** Flow cytometry was employed to show the effects of WWP2 knockdown alone or combined with CMTM6 overexpression on cell cycle. Data presented as mean ± SEM; * $P < 0.05$, **$P < 0.01$, *** $P < 0.001$, **** $P < 0.0001$; ns, not significant.
