# Supplementary material for: WWP2-induced inhibition of hepatocellular carcinoma cellular senescence via the ubiquitination and degradation of p21
Source: Cell Death Dis. 2025 Dec 12;17(1):96. doi: 10.1038/s41419-025-08318-0 (PMC12830804; doi:10.1038/s41419-025-08318-0)

Supplmentary meterial:Original Western Blots
Titlle: WWP2-Induced Inhibition of Hepatocellular Carcinoma Cellular Senescence via the Ubiquitination and Degradation of p21

Figure 1F

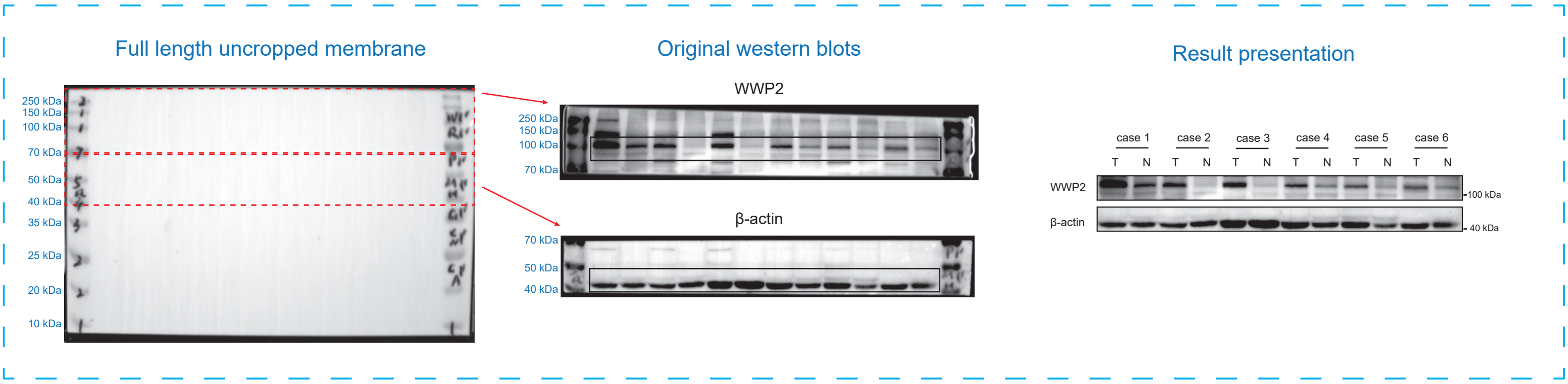

Figure 2C

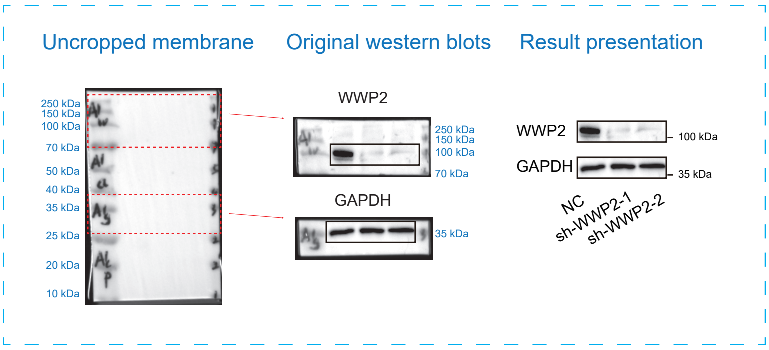

Figure 2D

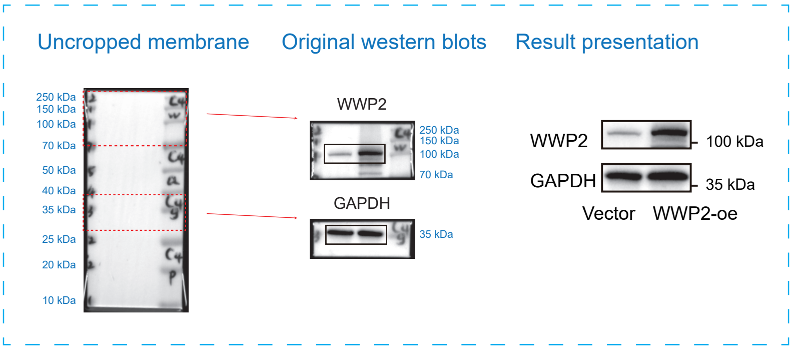

Figure 3G

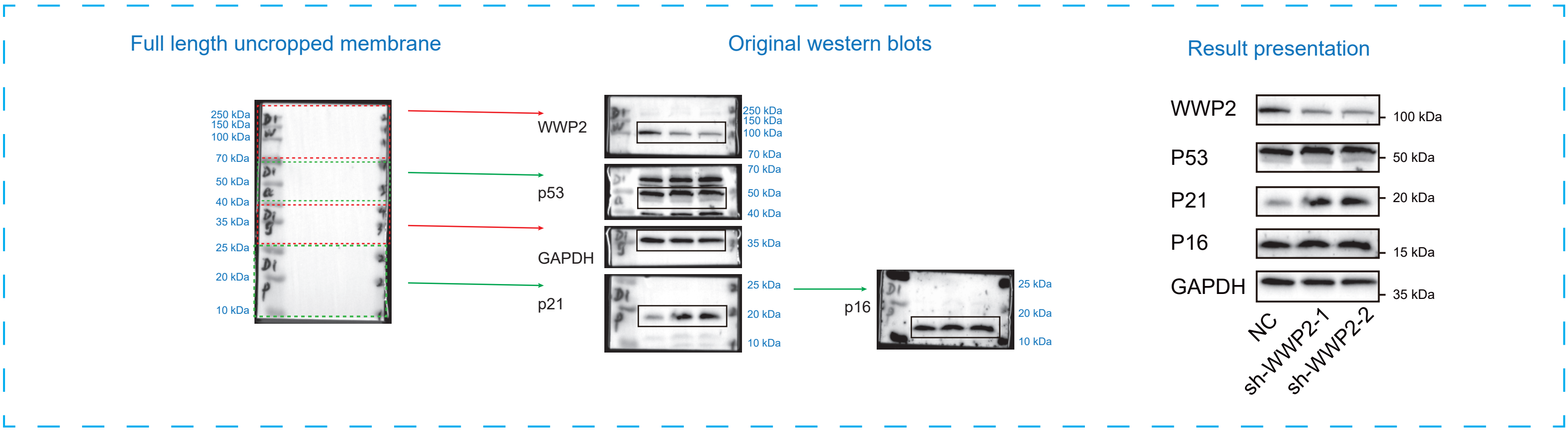

Figure 3H

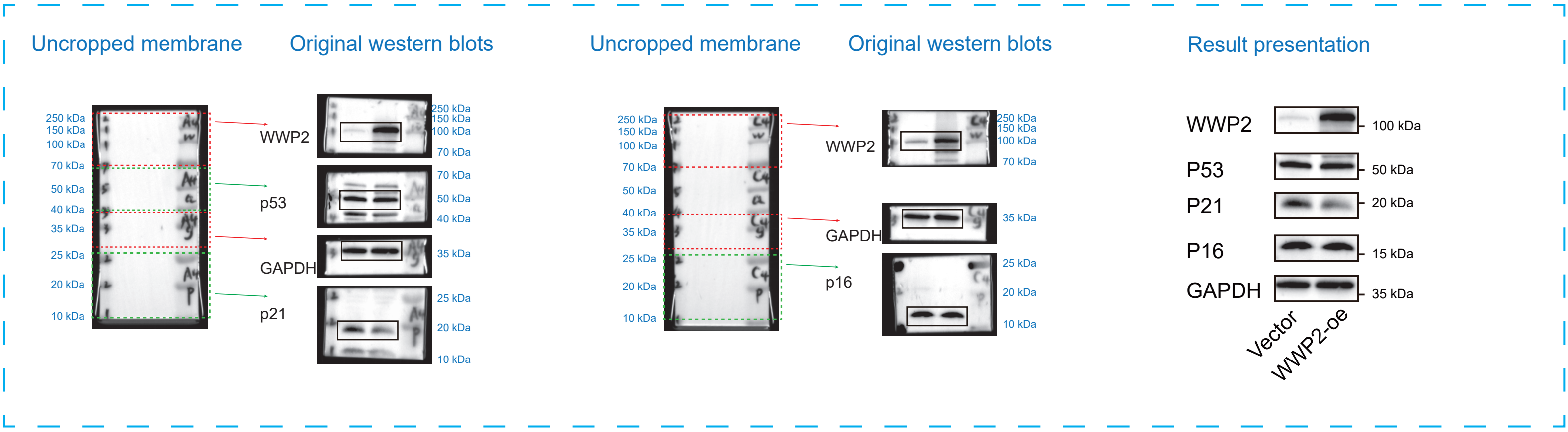

Figure 4B

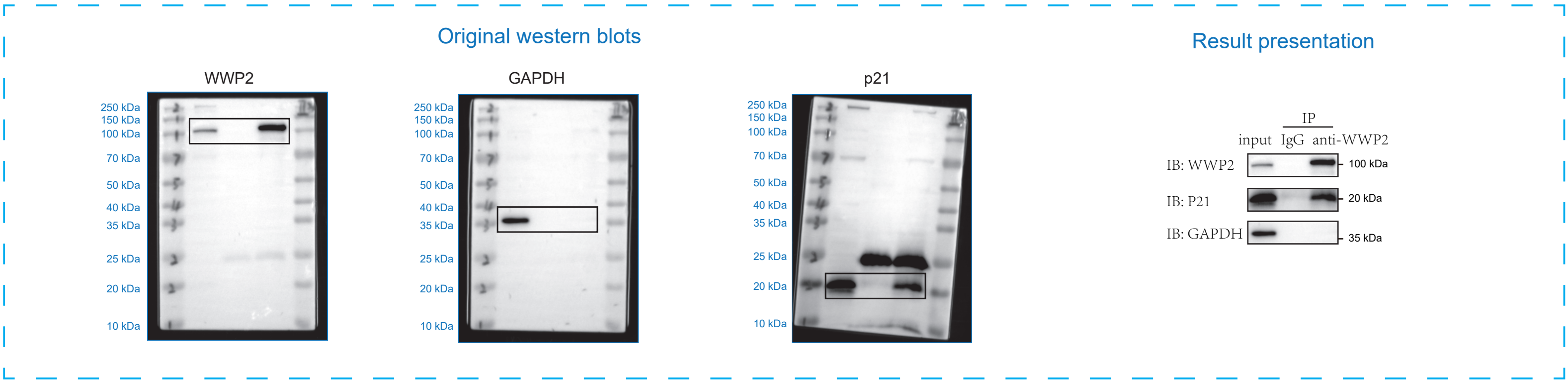

Figure 4C

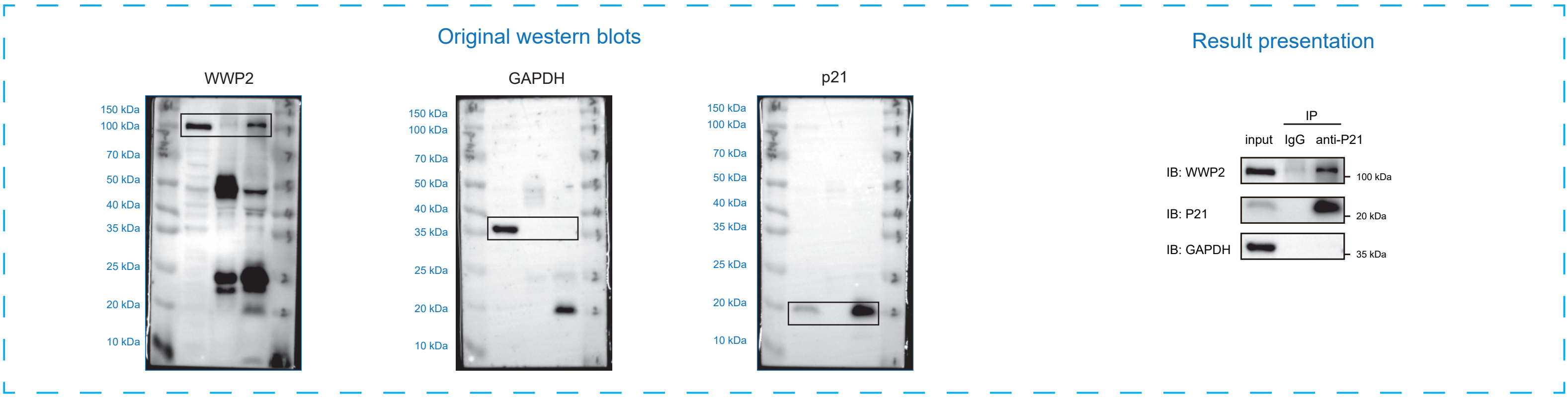

Figure 4D

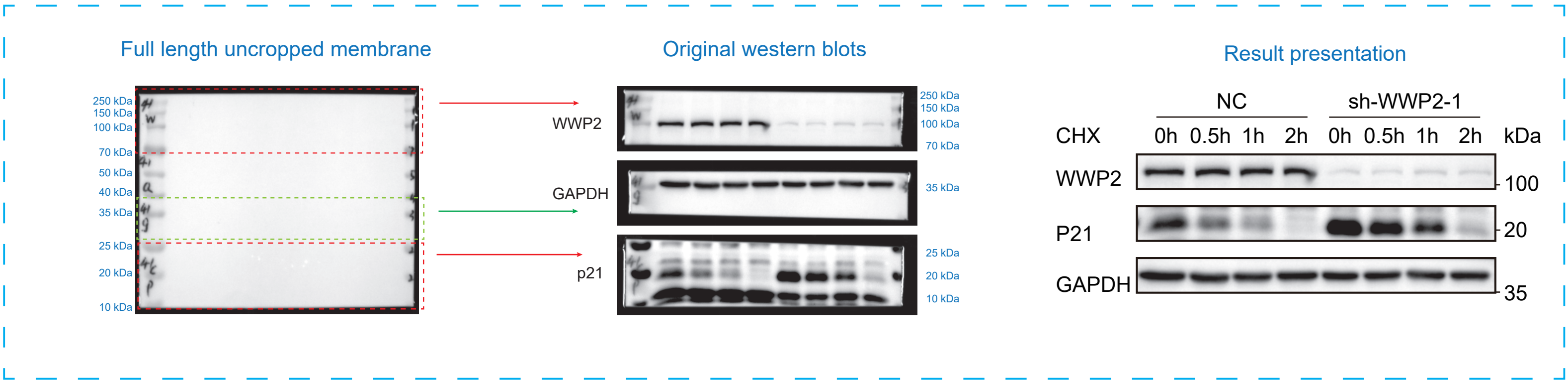

Figure 4E

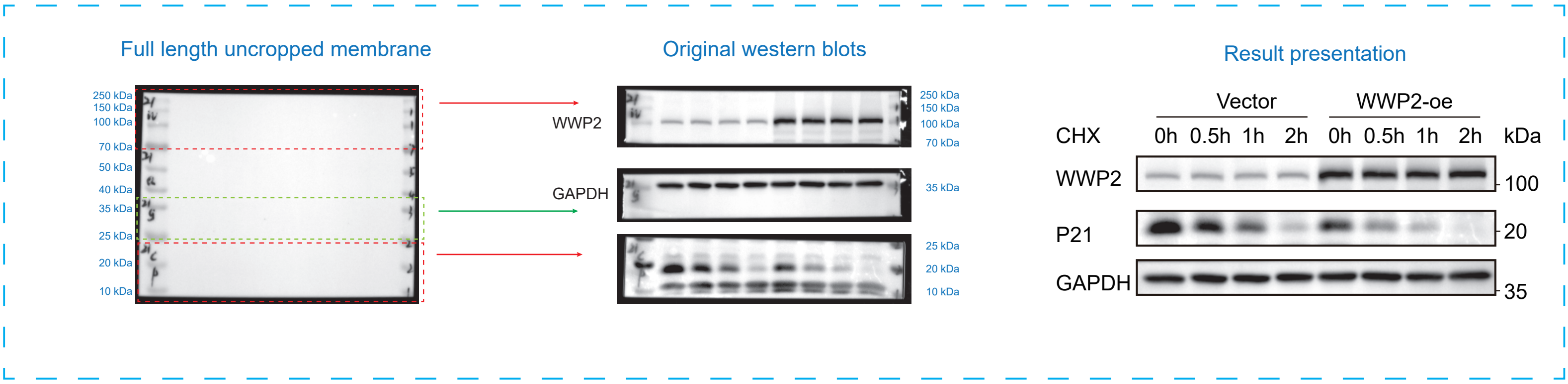

Figure 4F

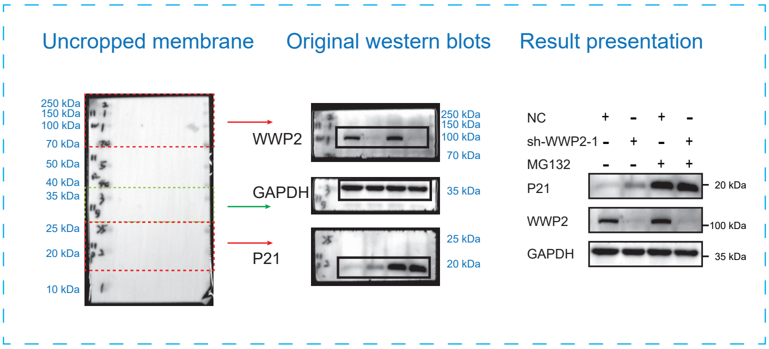

Figure 4G

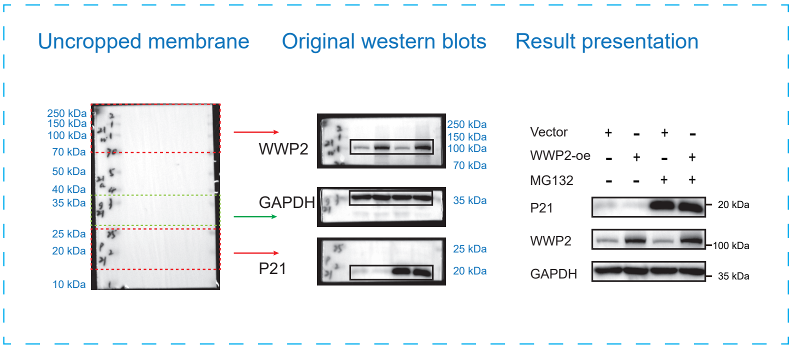

Figure 4H

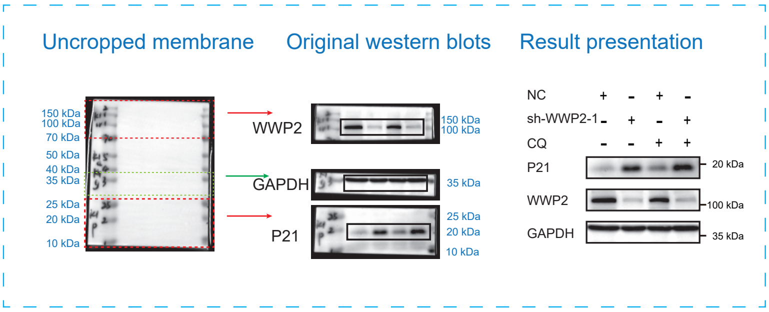

Figure 4I

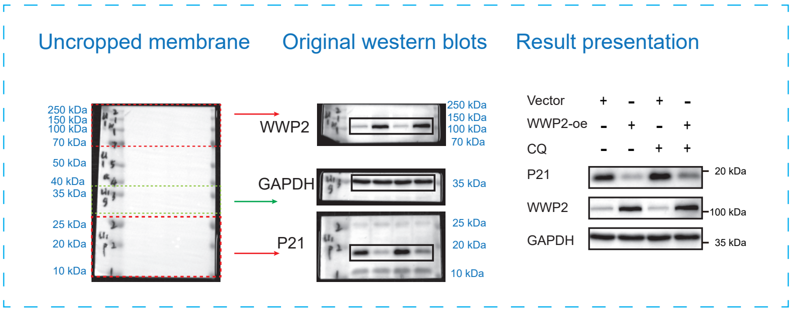

Figure 4J

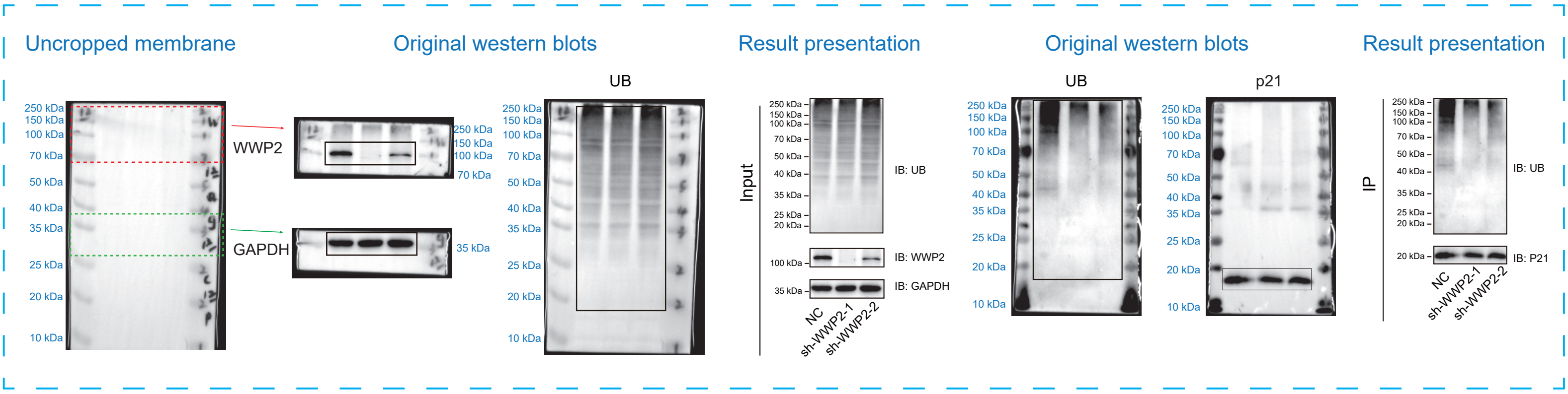

Figure 4K

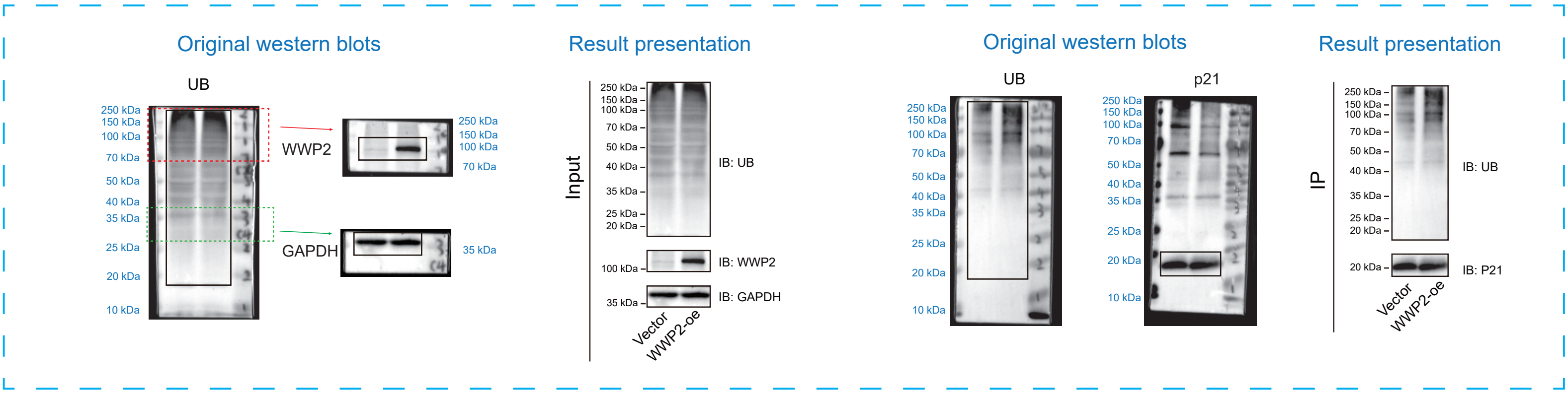

Figure 4L

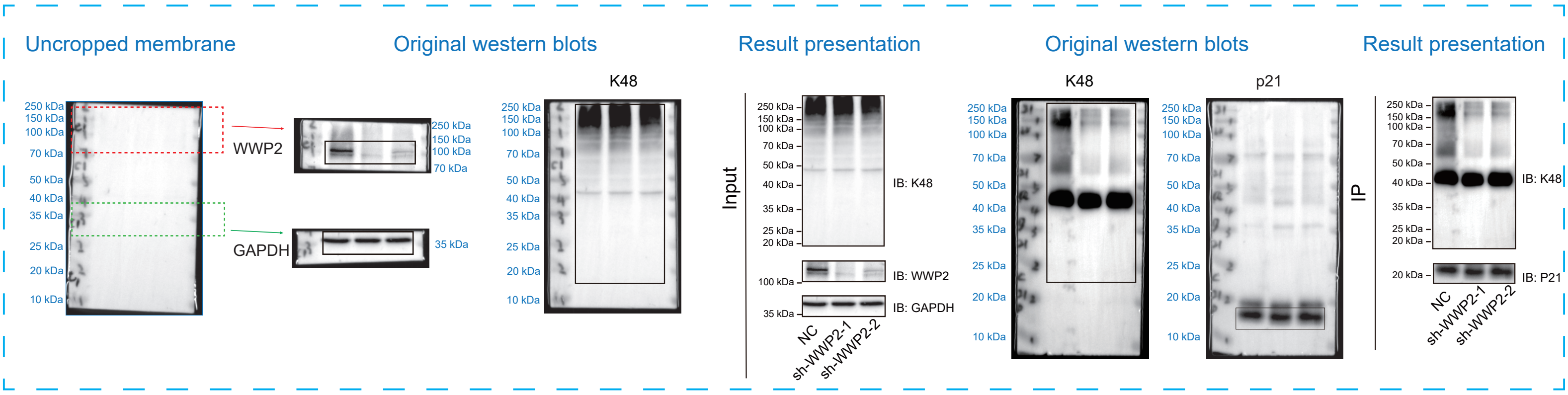

Figure 4M

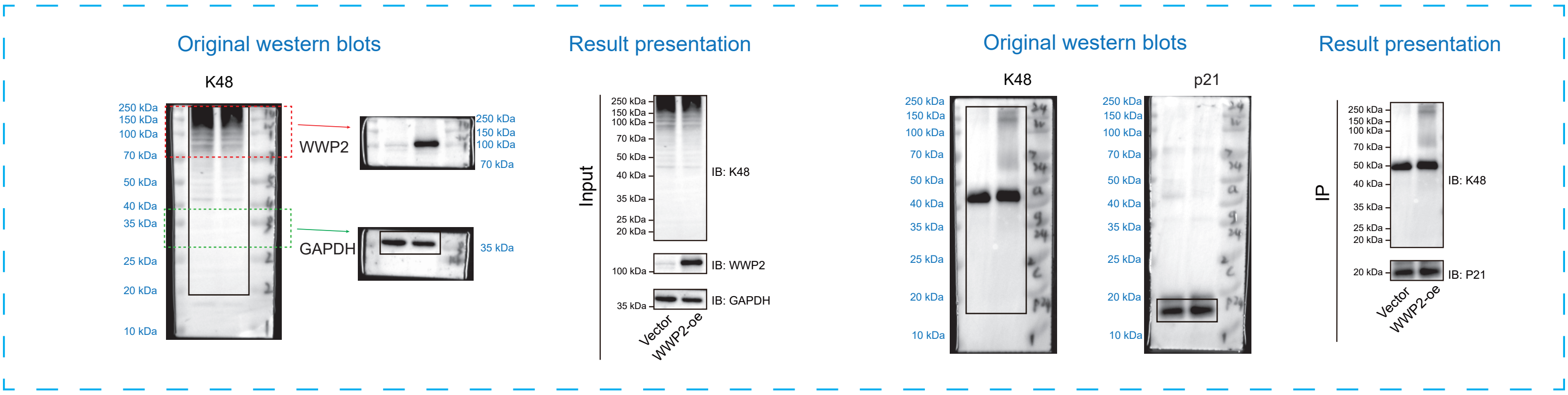

Figure 4N

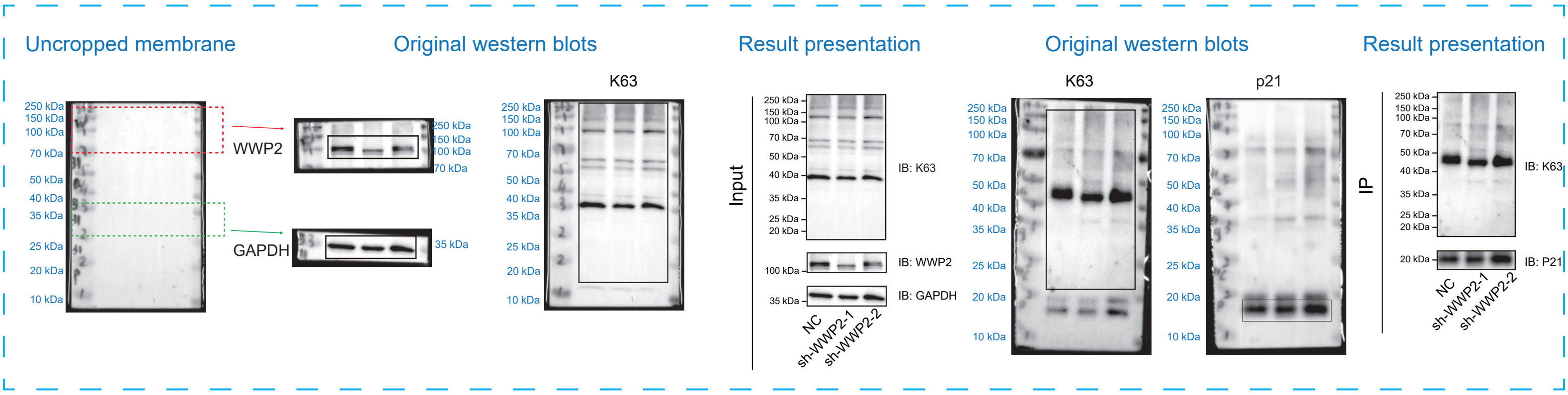

Figure 4O

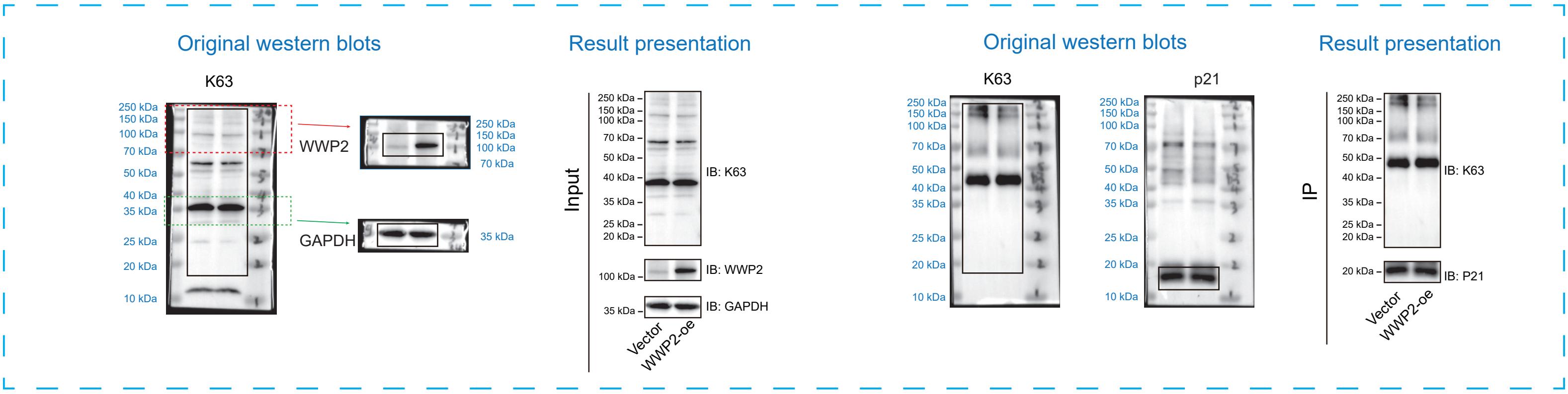

Figure 5B

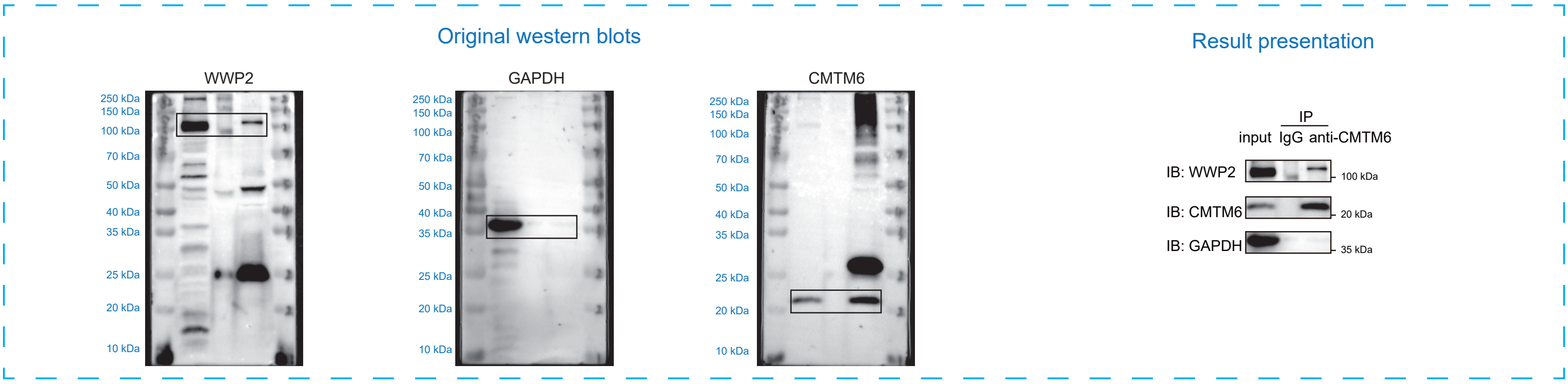

Figure 5C

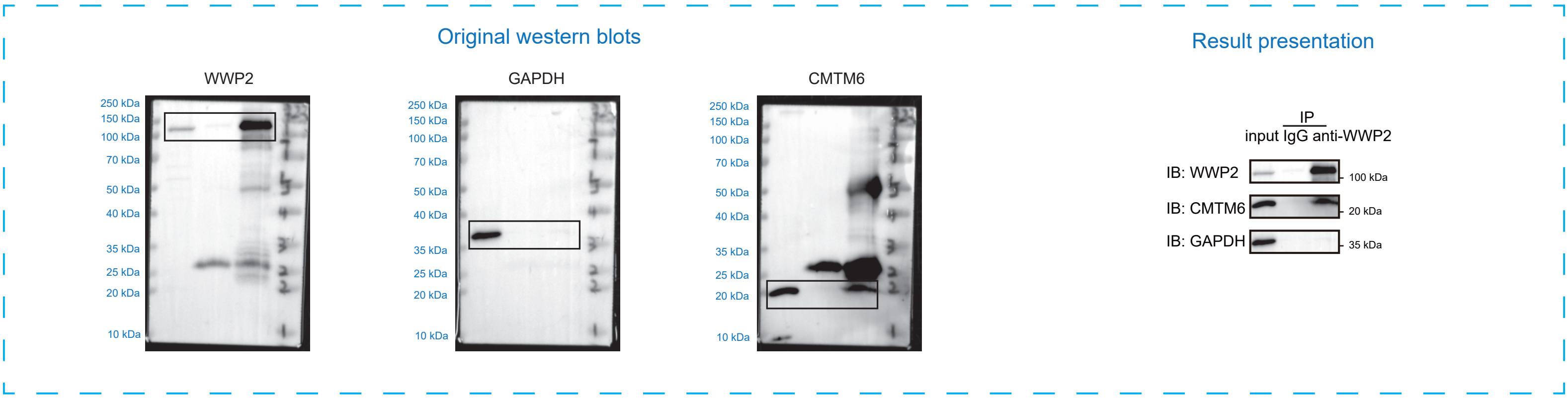

Figure 5E

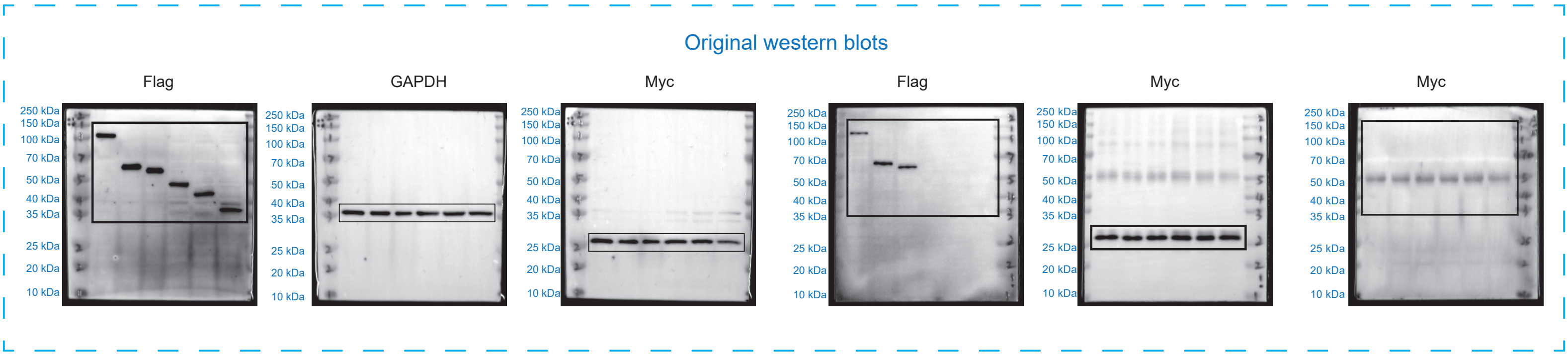

Figure 5G

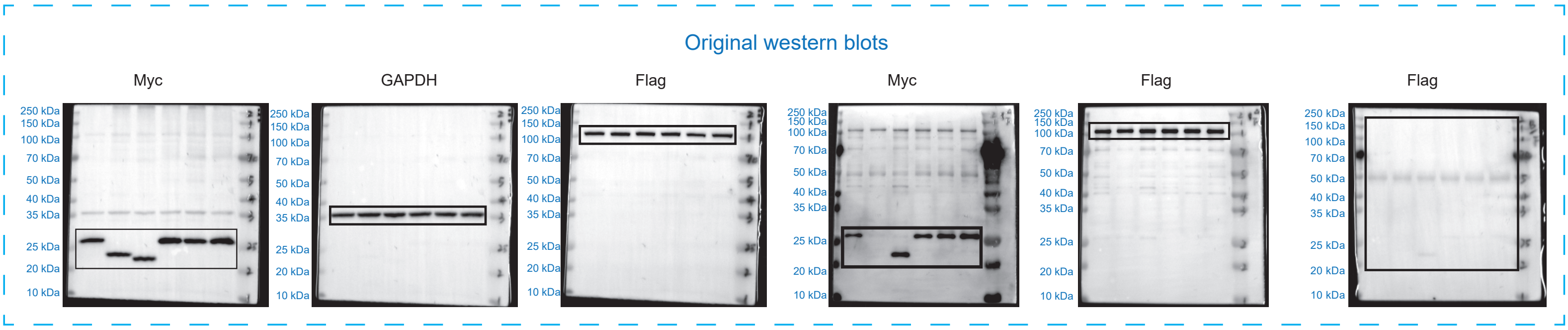

Figure 5H

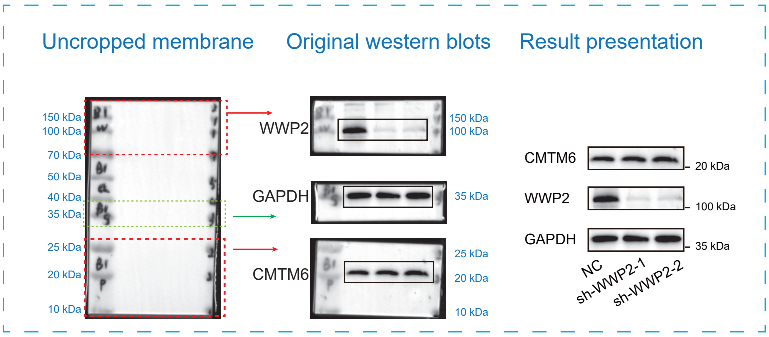

Figure 5I

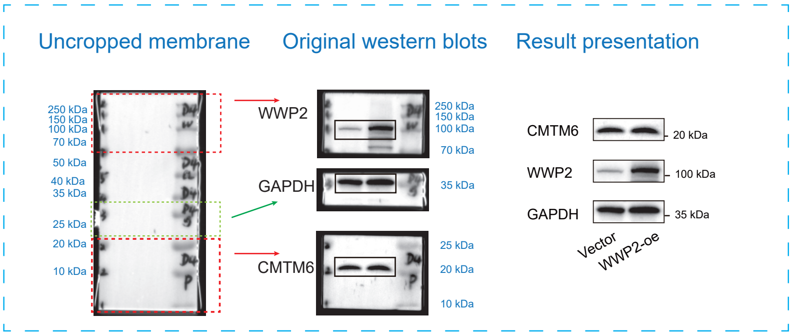

Figure 5J

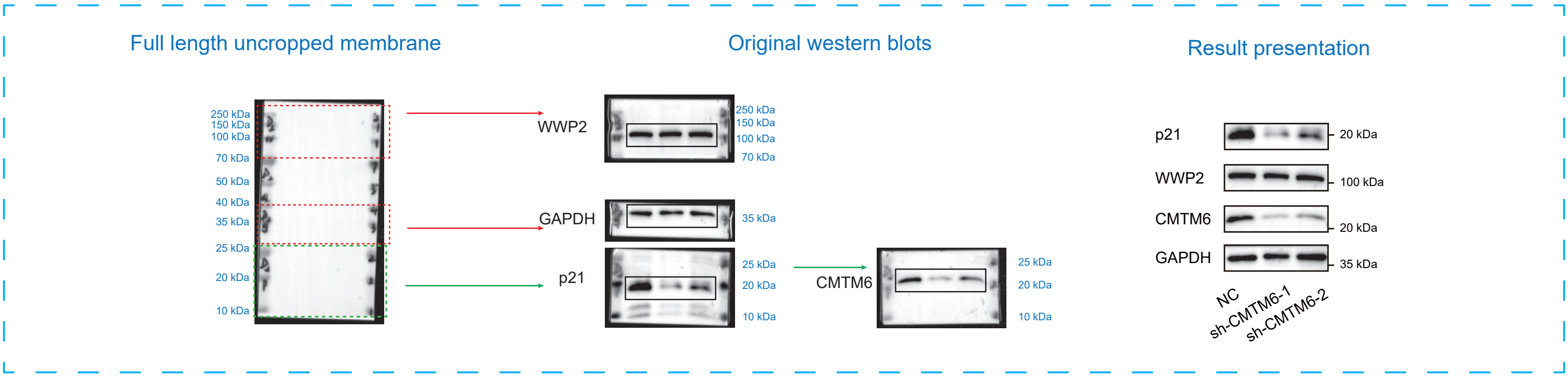

Figure 5K

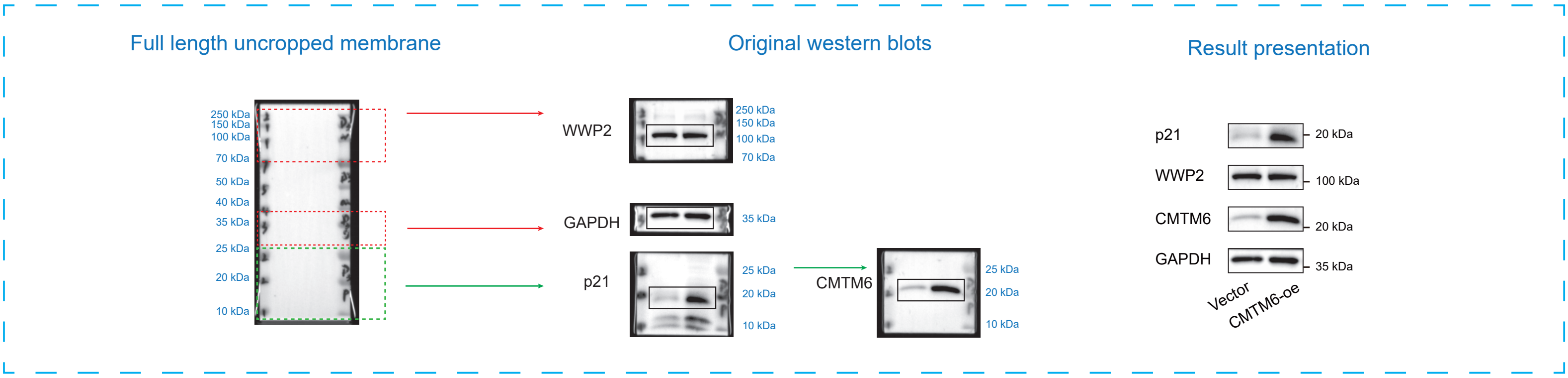

Figure 7A

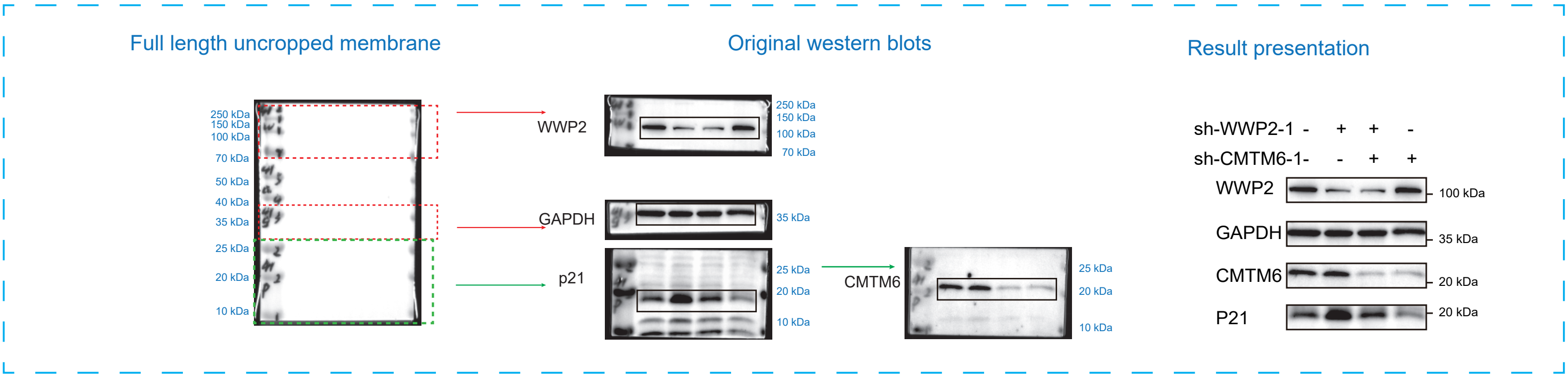

Figure 7B

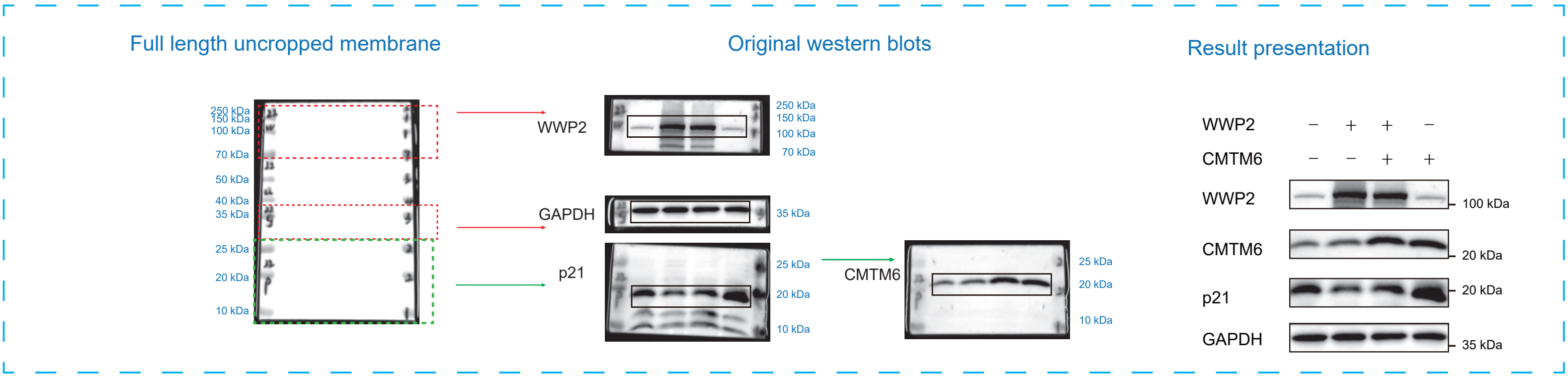

Figure 7C

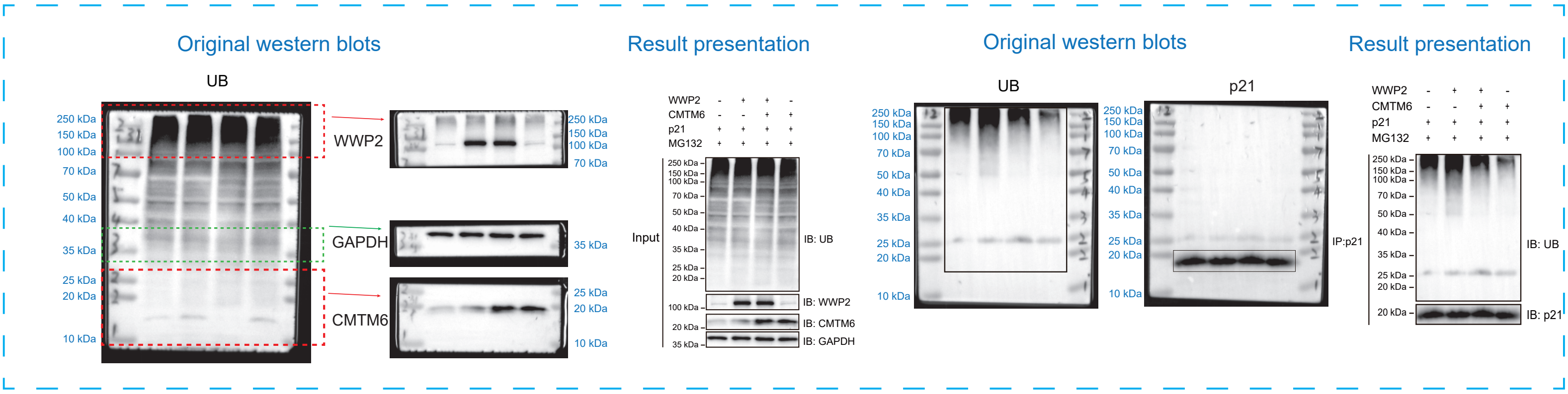

Figure 7D

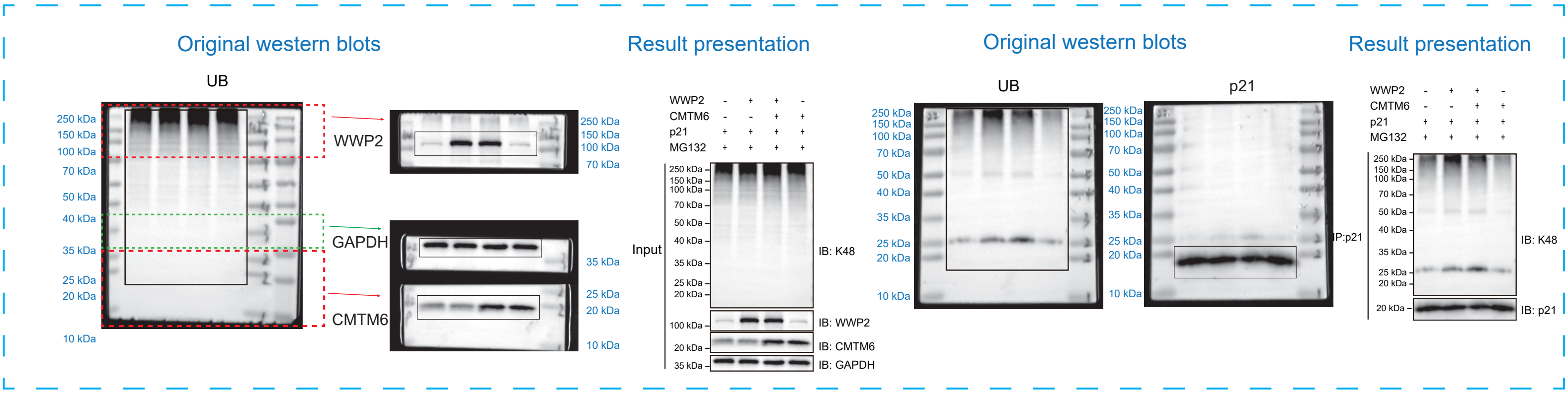

Figure 7E

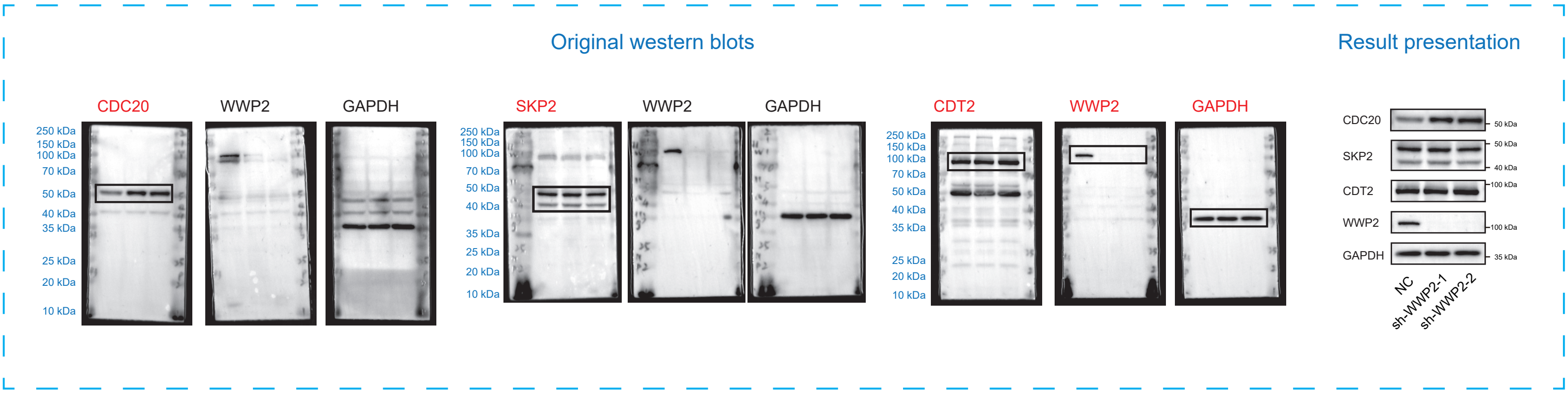

Figure 7F

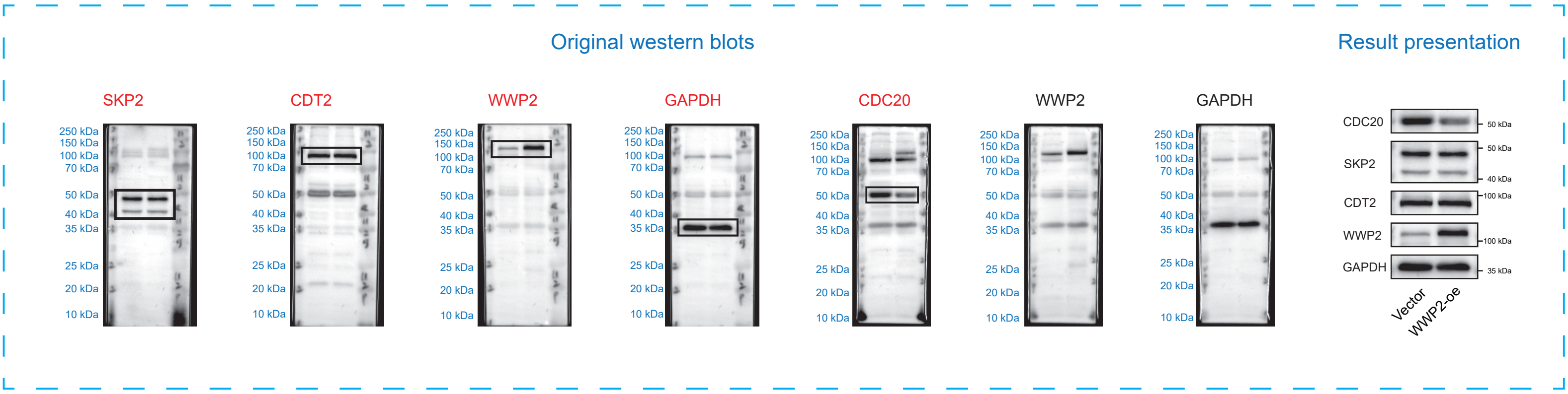

Figure 8G

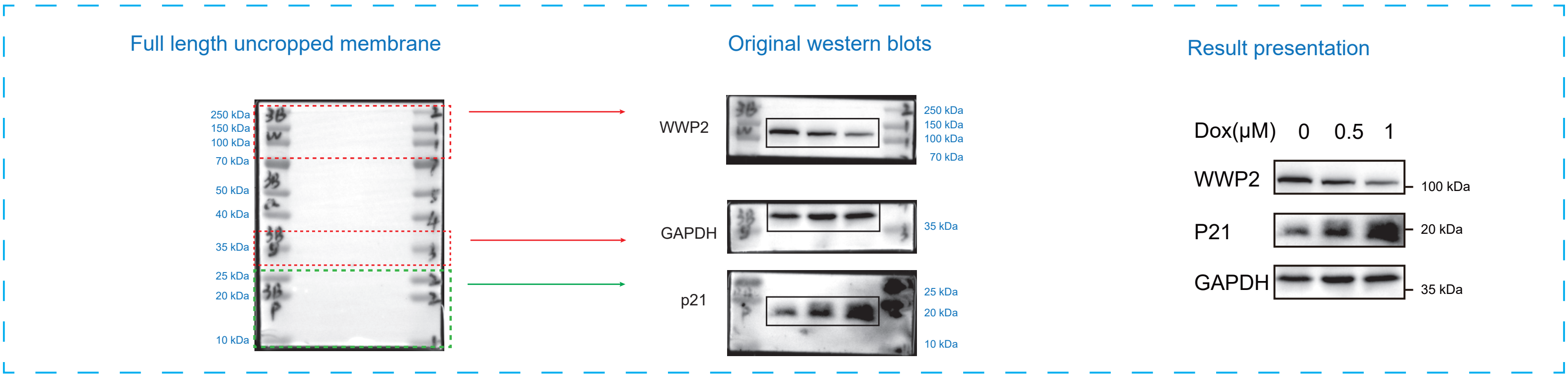

Figure 8H

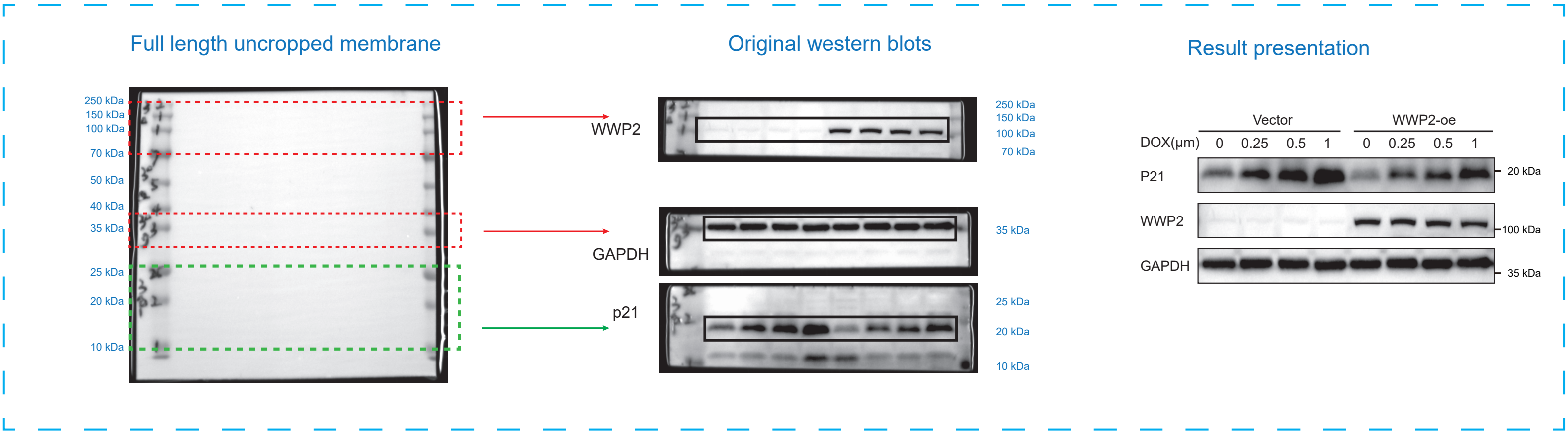

Supplementary Figure 1D

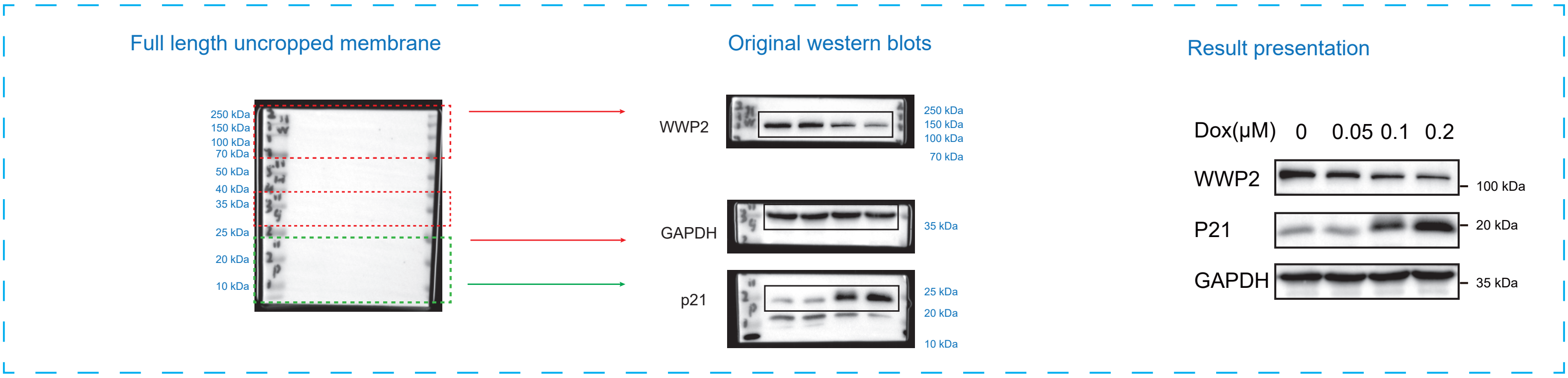

Supplementary Figure 1E

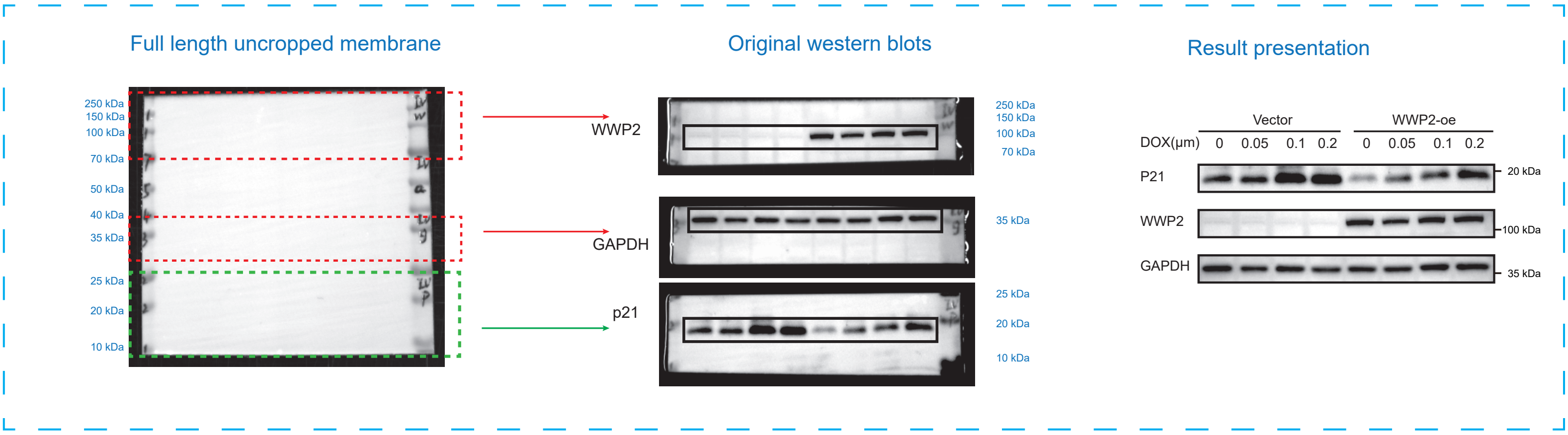

Supplementary Figure 2A

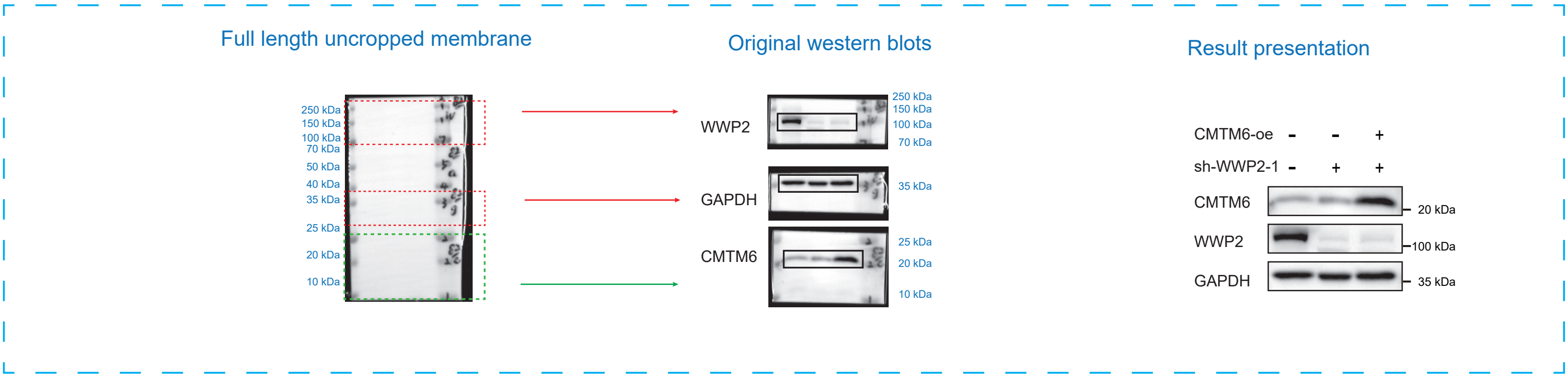

Supplement: Supplementary file 4 — Original Western Blots [file 41419_2025_8318_MOESM4_ESM.pdf]
